# Supplementary material for: Anthropogenic nutrient inputs cause excessive algal growth for nearly half the world’s population
Source: Nat Commun. 2025 Feb 20;16:1830. doi: 10.1038/s41467-025-57054-8 (PMC11842711; doi:10.1038/s41467-025-57054-8)
Supplement: Supplementary file 1 — Supplementary Information [file 41467_2025_57054_MOESM1_ESM.pdf]

Supplementary Information for:

Anthropogenic nutrient inputs cause excessive algal growth for nearly half the world's population

Richard W. McDowell[1,2], Dongwen Luo[3], Peter Pletnyakov[1], Martin Upsdell[3], Walter K. Dodds[4].*
[1]AgResearch, Lincoln Science Centre, Private Bag 4749, Christchurch 8140, New Zealand.
[2]Faculty of Agriculture and Life Sciences, P O Box 84, Lincoln University, Lincoln 7647, Christchurch, New Zealand.
[3]AgResearch, Ruakura Research Centre, 10 Bisley Road, Hamilton 3214, New Zealand
[4]Division of Biology, Kansas State University, Manhattan, KS 66506, USA
**Corresponding author's email: Richard.mcdowell@agresearch.co.nz*

The following text contains supplementary information for the methods and outputs in the main text. It is organised as a series of supplementary figures and tables either separately or in combination with supplementary notes that go into further detail.

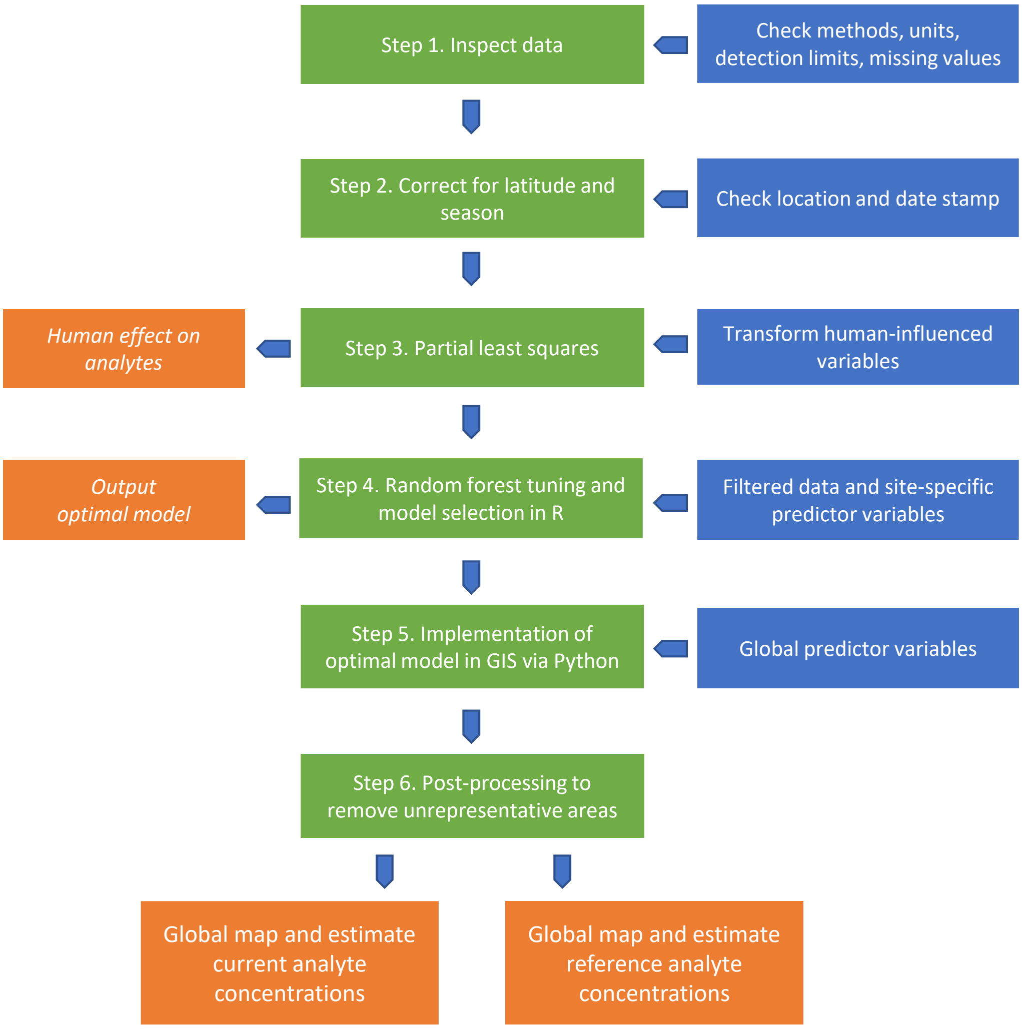

Supplementary Fig 1 showing the steps involved in filtering and processing the data to obtain the estimates of current and reference concentrations of each analyte.

Supplementary Table 1 showing the variables and their sources used to create a single human effect variable and considered in the final model to predict the concentrations of nitrogen and phosphorus fractions at each site. The short title for each variable used in the modelling process is given in parentheses.

Variable (years of data used)	Units	Source
Human effect variables		
Crop cover 2009 *(crop)*	% of catchment	1
Forest cover 2009 *(forest)*	% of catchment	1
Lentic cover 2009 *(lentic)*	% of catchment	1
Pasture cover 2009 *(pasture)*	% of catchment	1
Rangeland cover 2009 *(range_land)*	% of catchment	1
Urban cover 2009 *(urban)*	% of catchment	1
Soil Olsen P stock (2010) *(OlsenP)*	kg ha^{-1}	2
Population density (2000) *(PopDens)*	Number of people km^{-2}	3
Catchment characteristics considered in final models		
Human effect *(Human)*	mg L^{-1}	from above
Number of observations at each site *(NObs)*	-	-
Mean altitude (2008) *(Mean_Altitude)*	m above sea level	4
Mean slope (2008) *(Mean_Slope)*	%	4
Soil order (2015) *(S_order)*	% of catchment	5
GDP per capita (2015) *(GDP)*	USD $person^{-1}$	6
Latitude *(Latitude)*	Degrees	7
Catchment area (2008) *(Area_SqKm)*	km^2	4
Mean evapotranspiration (1950-1999) *(ET)*	mm yr^{-1}	8
Mean potential evapotranspiration (1950-1999) *(ETpot)*	mm yr^{-1}	8
Soil wetness (1950-1999) *(SoilWet)*	mm over profile	8
Mean daily runoff by month (1-12) (2000)[a] *(runoff_n)*	mm	9
Mean rainfall (1970-2000)[a] *(rainfall_n)*	mm yr^{-1}	10
Mean precipitation by month (1-12) (1970-2000) *(precip_n)*	mm	10
Ecoregion within biomes *(ECO_NAME)*	Categorical	11
Global ecological land units – Bioclimate *(EF_Bio_Des)*	Categorical	12
Global ecological land units – landform *(EF_LF_Desc)*	Categorical	12
Global ecological land units – lithology *(EF_Lit_Des)*	Categorical	12
Terrestrial biomes *(BIOME_NAME)*	Categorical	11

[a] Note that we adjusted the 12 variables measuring the average water runoff and rainfall in each of the 12 months of the year to account for seasonality. For sites in the northern hemisphere, the months were advanced by six. Hence for sites in the southern hemisphere, month 1 is January, while month 1 is July for sites in the northern hemisphere. "_n" refers to month (1-12) in the shortened name. Latitude was included in the model for two reasons: 1) to identify the split between the topics and seasonal data from temperate zones and 2) to act as a catch-all for the interaction of biophysical parameters.

Supplementary Note 1: Deriving Human Effects

We wished to derive one factor to account for human effects. We used partial least squares [13] to amalgamate anthropogenic effects on each analyte. Partial least squares avoided any potential effect from multi-collinearity amongst variables (especially land uses). The resulting distribution of human effects was normally distributed across each analyte (Supplementary Fig 2).

Olsen phosphorus was the most important predictor of human effect in dissolved reactive phosphorus and total nitrogen. Olsen phosphorus may capture the intensity of farm practices better than proportions of land use. For instance, nutrient losses from grassland can vary by orders of

magnitude depending on the fertility of the soils – as reflected by their Olsen phosphorus concentration [14]. Population density was the most important component of human effect in nitrate-nitrogen and total phosphorus. Population density could reflect a need to intensively produce food (with high nutrient losses) close to cities to avoid transportation costs but is probably more a reflection of point source discharges [15].

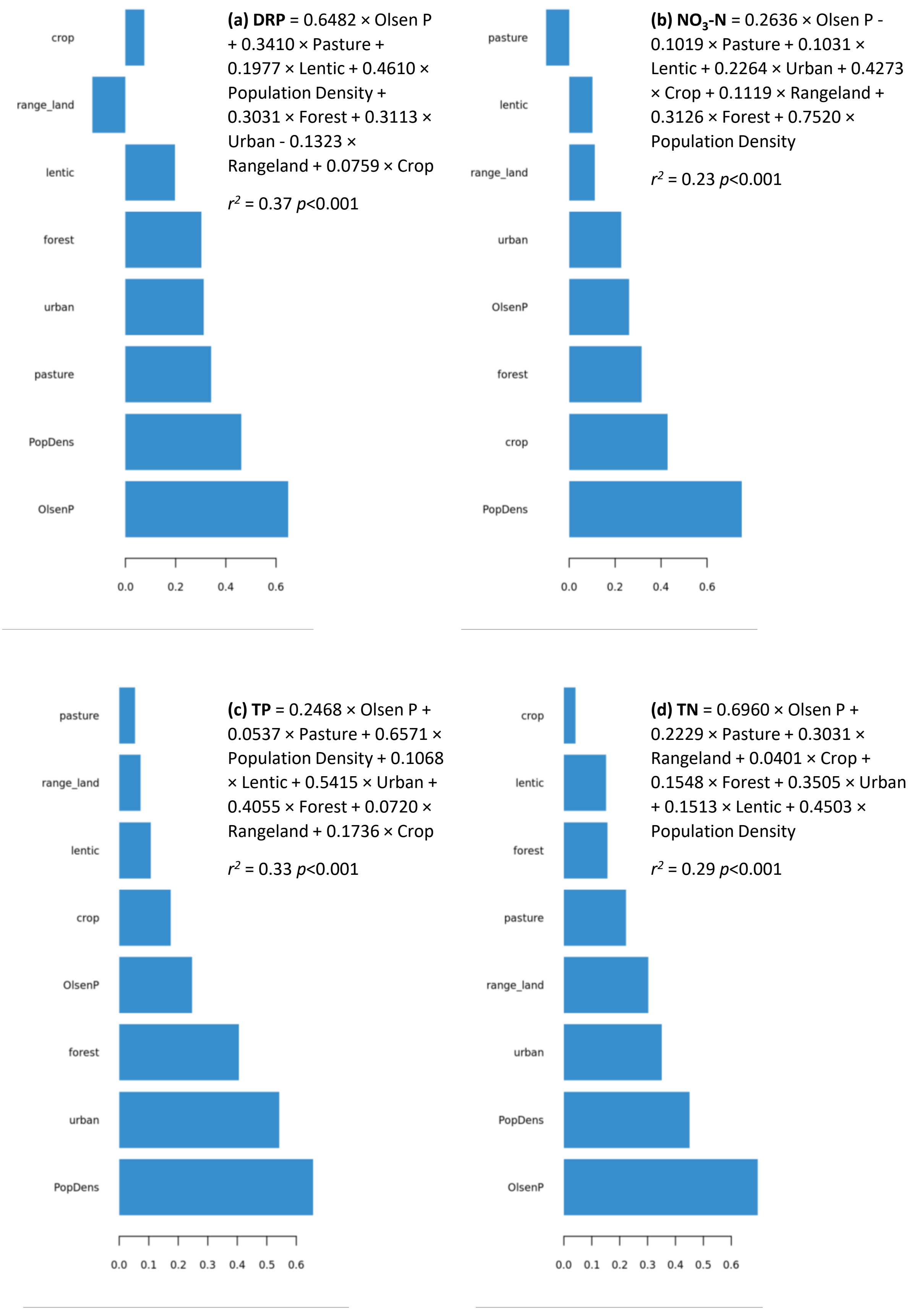

Supplementary Fig 2. Output of the partial least squares analysis of human effects for each analyte (a = DRP, dissolve reactive phosphorus; b = NO_3-N, nitrate-nitrogen; c = TP, total phosphorus; d = TN, total nitrogen) showing the relative importance of each predictor. The function is also given for

each human effect along with its coefficients and performance (coefficient of determination and significance).

Supplementary Note 2: Finding a suitable model in R

We modelled each analyte in R to derive suitable models after first examining the representativeness of the training and test data to the total dataset (Supplementary Table 2) and tuning the predictor variables, choosing the final model to be implemented in Python within ArcGIS Pro (the R models could not be implemented in ArcGIS Pro) based on a small mean absolute error, high coefficient of determination and the fewest terms (Supplementary Table 3, Supplementary Table 4, Supplementary Table 5, Supplementary Table 6). The performance of the final model was good [16] with a low mean absolute error and high coefficient of determination. Importantly, human effect was the most important predictor relative to all other predictors chosen in each model (Supplementary Fig 3, Supplementary Fig 4, Supplementary Fig 5, Supplementary Fig 6).

Supplementary Table 2 giving descriptive statistics (natural log) for the combined dataset and those split and used for training and testing the random forest models in R for each log-transformed nitrogen (NO_3-N = nitrate-nitrogen, TN = total nitrogen) and P (DRP = dissolved reactive phosphorus, TP = total phosphorus) fraction.

Parameter	Count	Mean	Median	Std deviation	25th percentile	75th percentile	Min	Max
DRP all	2022	-5.167	-5.307	0.042	-6.908	-3.685	-8.080	3.726
DRP train	1516	-5.164	-5.307	0.048	-6.908	-3.685	-8.080	3.726
DRP test	506	-5.176	-5.307	0.082	-6.908	-3.685	-8.080	0.642
NO_3-N all	2189	-1.492	-1.427	0.032	-2.408	-0.511	-6.475	3.059
NO_3-N train	1641	-1.488	-1.427	0.038	-2.408	-0.511	-6.475	2.708
NO_3-N test	548	-1.503	-1.427	0.064	-2.408	-0.511	-6.475	3.059
TP all	2438	-2.822	-2.781	0.032	-3.905	-1.833	-9.284	2.196
TP train	1827	-2.825	-2.781	0.036	-3.905	-1.833	-9.284	2.196
TP test	611	-2.814	-2.786	0.064	-3.905	-1.833	-5.809	1.435
TN all	1007	-0.820	-0.674	0.045	-2.038	0.095	-4.605	3.090
TN train	753	-0.819	-0.674	0.053	-2.038	0.086	-4.605	3.090
TN test	254	-0.824	-0.673	0.088	-2.022	0.095	-4.605	2.198

Supplementary Table 3 showing the array of initial models for the prediction of dissolved reactive phosphorus along with performance metrics as implemented in R. Also included are model tuning (predictor variables, j; trees, i; and nodes, k) and performance metrics (mean absolute error (MAE), root mean square error (RMSE), and the coefficient of determination (R^2)).

Model	i	j	k	Terms	MAE	RMSE	R2	Model terms
1	100	16	4	25	0.750	1.103	0.815	Latitude + Runoff_1 + Runoff_2 + Runoff_3 + Runoff_4 + Runoff_5 + Runoff_6 + Runoff_7 + Runoff_8 + Runoff_9 + Runoff_10 + Runoff_11 + Runoff_12 + Area_SqKm + Mean_Slope + S_Order + LC + BIOME_NAME + EF_Lit_Des + ET + ETPot + SoilWet + Bio_temp + Bio_Moist + human_effect
2	500	19	1	24	0.751	1.110	0.812	EF_Lit_Des + LC + Runoff_8 + Runoff_7 + Runoff_10 + S_Order + Runoff_4 + Runoff_9 + Runoff_12 + Runoff_1 + Runoff_11 + SoilWet + Runoff_2 + Runoff_6 + Area_SqKm + Runoff_5 + Bio_temp + Runoff_3 + ET + ETPot + Mean_Slope + Latitude + BIOME_NAME + human_effect
3	300	13	1	23	0.749	1.104	0.815	LC + Runoff_8 + Runoff_7 + Runoff_10 + Runoff_4 + Runoff_9 + S_Order + Runoff_1 + Runoff_11 + Runoff_12 + SoilWet + Runoff_2 + Bio_temp + Runoff_3 + Runoff_6 + Runoff_5 + Area_SqKm + ETPot + ET + Mean_Slope + Latitude + BIOME_NAME + human_effect
4	300	13	3	22	0.749	1.104	0.814	Runoff_8 + Runoff_7 + Runoff_10 + S_Order + Runoff_4 + Runoff_9 + Runoff_1 + SoilWet + Runoff_11 + Runoff_12 + Runoff_6 + Runoff_5 + Area_SqKm + Bio_temp + Runoff_2 + Runoff_3 + ETPot + ET + Mean_Slope + Latitude + BIOME_NAME + human_effect
5	500	13	1	21	0.750	1.106	0.813	Runoff_7 + Runoff_10 + Runoff_4 + S_Order + Runoff_9 + Runoff_1 + SoilWet + Runoff_12 + Runoff_11 + Runoff_6 + Runoff_5 + Runoff_2 + Area_SqKm + Bio_temp + Runoff_3 + ETPot + ET + Mean_Slope + Latitude + BIOME_NAME + human_effect
6	300	13	1	20	0.748	1.104	0.814	Runoff_4 + Runoff_7 + Runoff_1 + S_Order + Runoff_9 + Runoff_12 + SoilWet + Runoff_11 + Runoff_2 + Runoff_6 + Bio_temp + Runoff_3 + Runoff_5 + Area_SqKm + ETPot + ET + Mean_Slope + Latitude + BIOME_NAME + human_effect
7	300	13	3	19	0.746	1.101	0.815	Runoff_7 + S_Order + Runoff_1 + Runoff_11 + Runoff_12 + SoilWet + Runoff_9 + Bio_temp + Runoff_6 + Runoff_2 + Runoff_5 + Area_SqKm + Runoff_3 + ET + ETPot + Mean_Slope + Latitude + BIOME_NAME + human_effect
8	500	13	1	18	0.744	1.103	0.814	S_Order + Runoff_1 + Runoff_12 + Runoff_11 + Runoff_9 + Bio_temp + SoilWet + Runoff_2 + Area_SqKm + Runoff_6 + Runoff_5 + Runoff_3 + ETPot + ET + Mean_Slope + Latitude + BIOME_NAME + human_effect
9	300	11	3	17	0.742	1.100	0.815	Runoff_1 + SoilWet + Runoff_12 + Runoff_11 + Runoff_9 + Bio_temp + Runoff_2 + Runoff_5 + Runoff_6 + Area_SqKm + Runoff_3 + ETPot + ET + Mean_Slope + Latitude + BIOME_NAME + human_effect
10	900	9	1	16	0.744	1.097	0.817	Runoff_12 + SoilWet + Runoff_11 + Runoff_9 + Runoff_2 + Area_SqKm + Bio_temp + Runoff_6 + Runoff_5 + Runoff_3 + ETPot + ET + Mean_Slope + Latitude + BIOME_NAME + human_effect
**11**	**300**	**9**	**1**	**15**	**0.741**	**1.097**	**0.816**	**SoilWet + Runoff_9 + Runoff_12 + Bio_temp + Area_SqKm + Runoff_5 + Runoff_2 + Runoff_6 + Runoff_3 + ETPot + Mean_Slope + ET + Latitude + BIOME_NAME + human_effect**
12	100	7	4	14	0.740	1.093	0.818	Bio_temp + Runoff_9 + Runoff_12 + Area_SqKm + Runoff_6 + Runoff_3 + Runoff_2 + Runoff_5 + ETPot + ET + Mean_Slope + Latitude + BIOME_NAME + human_effect
13	700	7	1	13	0.749	1.105	0.813	Runoff_9 + Runoff_5 + Area_SqKm + Runoff_6 + Runoff_12 + Runoff_2 + Runoff_3 + ETPot + Mean_Slope + ET + Latitude + BIOME_NAME + human_effect
14	900	7	2	12	0.751	1.108	0.812	Area_SqKm + Runoff_12 + Runoff_6 + Runoff_5 + Runoff_2 + Runoff_3 + Mean_Slope + ETPot + ET + Latitude + BIOME_NAME + human_effect
15	900	6	2	11	0.752	1.106	0.813	Runoff_5 + Runoff_12 + Runoff_2 + Runoff_6 + Runoff_3 + Mean_Slope + ET + ETPot + Latitude + BIOME_NAME + human_effect
16	700	5	2	10	0.754	1.106	0.813	Runoff_5 + Runoff_6 + Runoff_2 + Runoff_3 + Mean_Slope + ET + ETPot + Latitude + BIOME_NAME + human_effect
17	500	4	2	9	0.752	1.101	0.815	Runoff_6 + Runoff_2 + Runoff_3 + Mean_Slope + ETPot + ET + Latitude + BIOME_NAME + human_effect
18	500	3	1	8	0.754	1.099	0.816	Runoff_6 + Runoff_3 + Mean_Slope + ET + ETPot + Latitude + BIOME_NAME + human_effect
19	500	3	1	7	0.752	1.098	0.816	Mean_Slope + Runoff_3 + ETPot + ET + Latitude + BIOME_NAME + human_effect
20	900	3	2	6	0.750	1.098	0.815	Runoff_3 + ET + ETPot + Latitude + BIOME_NAME + human_effect
21	900	3	2	5	0.769	1.124	0.806	ET + ETPot + Latitude + BIOME_NAME + human_effect
22	500	2	1	4	0.783	1.145	0.798	ETPot + Latitude + BIOME_NAME + human_effect
23	300	3	2	3	0.788	1.201	0.773	Latitude + BIOME_NAME + human_effect

Supplementary Table 4. **showing the array of initial models for the prediction of nitrate-nitrogen along with performance metrics as implemented in R.** Also included are model tuning (predictor variables, j; trees, i; and nodes, k) and performance metrics (mean absolute error (MAE), root mean square error (RMSE), and the coefficient of determination (R^2)).

Model	i	j	k	Terms	MAE	RMSE	R2	Model terms
1	500	7	4	25	0.728	0.991	0.762	Latitude + Runoff_1 + Runoff_2 + Runoff_3 + Runoff_4 + Runoff_5 + Runoff_6 + Runoff_7 + Runoff_8 + Runoff_9 + Runoff_10 + Runoff_11 + Runoff_12 + Area_SqKm + Mean_Slope + S_Order + LC + BIOME_NAME + EF_Lit_Des + ET + ETPot + SoilWet + Bio_temp + Bio_Moist + human_effect
2	700	7	1	24	0.729	0.991	0.761	EF_Lit_Des + Runoff_5 + Bio_temp + Runoff_3 + Runoff_6 + Runoff_2 + Runoff_7 + Runoff_4 + Runoff_10 + S_Order + Runoff_8 + Mean_Slope + Runoff_9 + Runoff_1 + SoilWet + Runoff_12 + Area_SqKm + ETPot + ET + LC + Runoff_11 + Latitude + BIOME_NAME + human_effect
3	500	7	3	23	0.728	0.989	0.762	Runoff_5 + Runoff_3 + Bio_temp + Runoff_7 + Runoff_6 + Runoff_4 + Runoff_2 + Runoff_10 + S_Order + Runoff_8 + Runoff_9 + Mean_Slope + SoilWet + Runoff_1 + Area_SqKm + Runoff_12 + ETPot + ET + LC + Runoff_11 + Latitude + BIOME_NAME + human_effect
4	300	7	1	22	0.728	0.990	0.762	Runoff_3 + Runoff_6 + Runoff_7 + Runoff_4 + Runoff_2 + Bio_temp + Runoff_10 + S_Order + Runoff_8 + Runoff_1 + Mean_Slope + Runoff_9 + SoilWet + Runoff_12 + Area_SqKm + ETPot + ET + LC + Runoff_11 + Latitude + BIOME_NAME + human_effect
**5**	500	7	1	21	0.729	0.991	0.761	Runoff_3 + Runoff_6 + Runoff_7 + Runoff_2 + Runoff_4 + Runoff_10 + Runoff_8 + S_Order + Runoff_1 + SoilWet + Runoff_9 + Mean_Slope + Runoff_12 + Area_SqKm + ETPot + LC + ET + Runoff_11 + Latitude + BIOME_NAME + human_effect
6	500	7	1	20	0.728	0.991	0.761	Runoff_6 + Runoff_7 + Runoff_2 + Runoff_4 + Runoff_10 + S_Order + Runoff_8 + Runoff_1 + Runoff_9 + Mean_Slope + SoilWet + Runoff_12 + Area_SqKm + ET + ETPot + LC + Runoff_11 + Latitude + BIOME_NAME + human_effect
7	300	7	1	19	0.729	0.991	0.761	Runoff_6 + Runoff_10 + Runoff_2 + Runoff_4 + S_Order + Runoff_8 + Runoff_9 + Runoff_1 + Mean_Slope + SoilWet + Runoff_12 + Area_SqKm + ETPot + ET + LC + Runoff_11 + Latitude + BIOME_NAME + human_effect
8	500	5	3	18	0.727	0.987	0.764	Runoff_6 + Runoff_2 + Runoff_4 + S_Order + Runoff_8 + Runoff_1 + Mean_Slope + Runoff_9 + SoilWet + Runoff_12 + Area_SqKm + ETPot + ET + LC + Runoff_11 + Latitude + BIOME_NAME + human_effect
9	700	5	1	17	0.727	0.988	0.763	Runoff_2 + S_Order + Runoff_4 + Runoff_8 + Runoff_1 + Mean_Slope + Runoff_9 + SoilWet + Area_SqKm + Runoff_12 + ETPot + ET + LC + Runoff_11 + Latitude + BIOME_NAME + human_effect
10	700	5	1	16	0.727	0.987	0.764	S_Order + Runoff_4 + Runoff_8 + Mean_Slope + Runoff_1 + Runoff_9 + SoilWet + Area_SqKm + Runoff_12 + ETPot + LC + ET + Runoff_11 + Latitude + BIOME_NAME + human_effect
11	300	5	1	15	0.733	0.995	0.759	Runoff_4 + Runoff_8 + Mean_Slope + Runoff_1 + Runoff_9 + SoilWet + Area_SqKm + Runoff_12 + ETPot + ET + LC + Runoff_11 + Latitude + BIOME_NAME + human_effect
12	300	5	4	14	0.733	0.993	0.760	Runoff_8 + Mean_Slope + Runoff_1 + SoilWet + Runoff_9 + Runoff_12 + Area_SqKm + ETPot + ET + LC + Runoff_11 + Latitude + BIOME_NAME + human_effect
**13**	**300**	**5**	**1**	**13**	**0.732**	**0.994**	**0.759**	**Mean_Slope + Runoff_1 + SoilWet + Runoff_9 + Runoff_12 + Area_SqKm + ETPot + ET + LC + Runoff_11 + Latitude + BIOME_NAME + human_effect**
14	300	5	1	12	0.731	0.996	0.757	Runoff_1 + SoilWet + Runoff_9 + Area_SqKm + Runoff_12 + ETPot + LC + ET + Runoff_11 + Latitude + BIOME_NAME + human_effect
15	500	3	1	11	0.731	0.992	0.761	SoilWet + Runoff_12 + Runoff_9 + ETPot + Area_SqKm + LC + ET + Runoff_11 + Latitude + BIOME_NAME + human_effect
16	300	3	3	10	0.734	0.994	0.759	Area_SqKm + Runoff_9 + Runoff_12 + ETPot + LC + ET + Runoff_11 + Latitude + BIOME_NAME + human_effect
17	500	2	2	9	0.743	1.000	0.755	Runoff_9 + LC + Runoff_12 + ETPot + ET + Runoff_11 + Latitude + BIOME_NAME + human_effect
18	100	2	2	8	0.743	1.004	0.753	LC + ETPot + Runoff_12 + ET + Runoff_11 + Latitude + BIOME_NAME + human_effect
19	900	2	1	7	0.745	1.008	0.750	ETPot + Runoff_12 + ET + Runoff_11 + Latitude + BIOME_NAME + human_effect
20	900	2	3	6	0.746	1.008	0.750	ETPot + ET + Runoff_11 + Latitude + BIOME_NAME + human_effect
21	500	2	1	5	0.745	1.012	0.748	ET + Runoff_11 + Latitude + BIOME_NAME + human_effect
22	500	2	2	4	0.757	1.017	0.744	ET + Latitude + BIOME_NAME + human_effect
23	700	2	2	3	0.806	1.080	0.707	Latitude + BIOME_NAME + human_effect

Supplementary Table 5. **showing the array of initial models for the prediction of total phosphorus along with performance metrics as implemented in R.** Also included are model tuning (predictor variables, j; trees, i; and nodes, k) and performance metrics (mean absolute error (MAE), root mean square error (RMSE), and the coefficient of determination (R^2)).

Model	i	j	k	Terms	MAE	RMSE	R2	Model terms
1	300	16	4	25	0.728	1.015	0.759	Latitude + Runoff_1 + Runoff_2 + Runoff_3 + Runoff_4 + Runoff_5 + Runoff_6 + Runoff_7 + Runoff_8 + Runoff_9 + Runoff_10 + Runoff_11 + Runoff_12 + Area_SqKm + Mean_Slope + S_Order + LC + BIOME_NAME + EF_Lit_Des + ET + ETPot + SoilWet + Bio_temp + Bio_Moist + human_effect
2	500	16	1	24	0.728	1.017	0.758	Runoff_6 + Runoff_1 + Runoff_10 + LC + S_Order + EF_Lit_Des + Runoff_8 + Runoff_4 + Runoff_11 + Runoff_3 + Runoff_2 + SoilWet + Runoff_12 + Runoff_7 + ET + Runoff_9 + Bio_temp + Mean_Slope + Area_SqKm + Runoff_5 + ETPot + Latitude + BIOME_NAME + human_effect
3	500	16	3	23	0.728	1.014	0.759	LC + Runoff_10 + Runoff_1 + S_Order + EF_Lit_Des + Runoff_4 + Runoff_8 + Runoff_3 + Runoff_11 + Runoff_2 + SoilWet + Runoff_12 + ET + Runoff_7 + Runoff_9 + Mean_Slope + Bio_temp + Area_SqKm + Runoff_5 + ETPot + Latitude + BIOME_NAME + human_effect
4	300	13	3	22	0.728	1.016	0.758	LC + Runoff_1 + S_Order + Runoff_4 + EF_Lit_Des + Runoff_8 + Runoff_11 + Runoff_3 + Runoff_2 + Runoff_12 + SoilWet + ET + Runoff_7 + Runoff_9 + Mean_Slope + Bio_temp + Area_SqKm + Runoff_5 + ETPot + Latitude + BIOME_NAME + human_effect
5	300	10	3	21	0.725	1.011	0.761	Runoff_1 + S_Order + EF_Lit_Des + Runoff_8 + Runoff_4 + Runoff_2 + Runoff_11 + Runoff_12 + Runoff_3 + SoilWet + ET + Runoff_9 + Mean_Slope + Runoff_7 + Bio_temp + Area_SqKm + Runoff_5 + ETPot + Latitude + BIOME_NAME + human_effect
6	900	13	3	20	0.731	1.020	0.756	Runoff_1 + S_Order + Runoff_11 + Runoff_4 + Runoff_8 + Runoff_12 + Runoff_3 + Runoff_2 + SoilWet + Runoff_9 + ET + Runoff_7 + Mean_Slope + Runoff_5 + Area_SqKm + Bio_temp + ETPot + Latitude + BIOME_NAME + human_effect
7	900	13	1	19	0.731	1.019	0.756	S_Order + Runoff_4 + Runoff_3 + Runoff_8 + Runoff_2 + Runoff_12 + Runoff_11 + SoilWet + ET + Runoff_7 + Runoff_9 + Bio_temp + Mean_Slope + Area_SqKm + Runoff_5 + ETPot + Latitude + BIOME_NAME + human_effect
8	300	13	1	18	0.732	1.022	0.755	Runoff_4 + Runoff_8 + Runoff_3 + Runoff_2 + Runoff_11 + SoilWet + Runoff_12 + ET + Runoff_7 + Runoff_9 + Bio_temp + Mean_Slope + Area_SqKm + Runoff_5 + ETPot + Latitude + BIOME_NAME + human_effect
9	700	9	1	17	0.732	1.021	0.756	Runoff_3 + Runoff_8 + Runoff_11 + SoilWet + Runoff_2 + Runoff_12 + ET + Runoff_7 + Bio_temp + Runoff_9 + Mean_Slope + Area_SqKm + Runoff_5 + ETPot + Latitude + BIOME_NAME + human_effect
10	700	11	1	16	0.732	1.022	0.755	Runoff_11 + SoilWet + Runoff_12 + Runoff_2 + Runoff_3 + ET + Runoff_9 + Bio_temp + Runoff_7 + Mean_Slope + Area_SqKm + Runoff_5 + ETPot + Latitude + BIOME_NAME + human_effect
11	300	7	3	15	0.731	1.018	0.758	Runoff_11 + Runoff_2 + SoilWet + Runoff_12 + ET + Bio_temp + Runoff_7 + Runoff_9 + Mean_Slope + Area_SqKm + Runoff_5 + ETPot + Latitude + BIOME_NAME + human_effect
12	500	9	2	14	0.731	1.020	0.756	Runoff_12 + SoilWet + ET + Runoff_2 + Bio_temp + Runoff_9 + Mean_Slope + Area_SqKm + Runoff_7 + Runoff_5 + ETPot + Latitude + BIOME_NAME + human_effect
13	700	7	1	13	0.730	1.018	0.757	ET + Runoff_12 + Runoff_2 + Bio_temp + Runoff_7 + Mean_Slope + Runoff_9 + Area_SqKm + Runoff_5 + ETPot + Latitude + BIOME_NAME + human_effect
**14**	**300**	**5**	**2**	**12**	**0.728**	**1.015**	**0.759**	**Runoff_2 + ET + Bio_temp + Mean_Slope + Runoff_9 + Area_SqKm + Runoff_7 + Runoff_5 + ETPot + Latitude + BIOME_NAME + human_effect**
15	100	6	5	11	0.731	1.021	0.755	Runoff_2 + ET + Mean_Slope + Runoff_9 + Area_SqKm + Runoff_7 + Runoff_5 + ETPot + Latitude + BIOME_NAME + human_effect
16	300	4	1	10	0.732	1.021	0.755	Mean_Slope + ET + Area_SqKm + Runoff_9 + Runoff_7 + Runoff_5 + ETPot + Latitude + BIOME_NAME + human_effect
17	900	4	2	9	0.734	1.024	0.753	Area_SqKm + Mean_Slope + Runoff_9 + Runoff_7 + Runoff_5 + ETPot + Latitude + BIOME_NAME + human_effect
18	300	3	1	8	0.737	1.023	0.754	Area_SqKm + Runoff_9 + Runoff_7 + Runoff_5 + ETPot + Latitude + BIOME_NAME + human_effect
19	700	3	1	7	0.740	1.026	0.752	Area_SqKm + Runoff_7 + Runoff_5 + ETPot + Latitude + BIOME_NAME + human_effect
20	700	3	2	6	0.745	1.032	0.748	Runoff_5 + Runoff_7 + ETPot + Latitude + BIOME_NAME + human_effect
21	100	3	1	5	0.751	1.041	0.742	Runoff_7 + ETPot + Latitude + BIOME_NAME + human_effect
22	300	2	3	4	0.771	1.057	0.733	ETPot + Latitude + BIOME_NAME + human_effect
23	100	2	3	3	0.818	1.105	0.705	Latitude + BIOME_NAME + human_effect

Supplementary Table 6. **showing the array of initial models for the prediction of total nitrogen along with performance metrics as implemented in R.** Also included are model tuning (predictor variables, j; trees, i; and nodes, k) and performance metrics (mean absolute error (MAE), root mean square error (RMSE), and the coefficient of determination (R^2)).

Model	i	j	k	Terms	MAE	RMSE	R2	Model terms
1	100	16	7	25	0.669	0.986	0.729	Latitude + Runoff_1 + Runoff_2 + Runoff_3 + Runoff_4 + Runoff_5 + Runoff_6 + Runoff_7 + Runoff_8 + Runoff_9 + Runoff_10 + Runoff_11 + Runoff_12 + Area_SqKm + Mean_Slope + S_Order + LC + BIOME_NAME + EF_Lit_Des + ET + ETPot + SoilWet + Bio_temp + Bio_Moist + human_effect
2	100	13	7	24	0.672	0.993	0.725	EF_Lit_Des + LC + Runoff_6 + Runoff_9 + S_Order + Runoff_10 + Runoff_12 + Runoff_8 + Bio_temp + Runoff_7 + Runoff_11 + SoilWet + Runoff_4 + Runoff_1 + Runoff_5 + ETPot + Mean_Slope + Area_SqKm + ET + Runoff_2 + Runoff_3 + Latitude + BIOME_NAME + human_effect
3	500	10	1	23	0.672	1.000	0.718	LC + S_Order + Runoff_6 + Runoff_12 + Runoff_11 + Runoff_10 + Runoff_9 + Runoff_7 + Runoff_8 + SoilWet + Bio_temp + Runoff_1 + Runoff_5 + Mean_Slope + Runoff_4 + ETPot + Area_SqKm + ET + Runoff_2 + Runoff_3 + Latitude + BIOME_NAME + human_effect
4	300	13	3	22	0.672	0.997	0.720	S_Order + Runoff_6 + Runoff_7 + Runoff_10 + Runoff_9 + Runoff_12 + Bio_temp + Runoff_8 + Runoff_11 + SoilWet + Runoff_1 + Runoff_5 + Mean_Slope + Area_SqKm + ETPot + ET + Runoff_4 + Runoff_2 + Runoff_3 + Latitude + BIOME_NAME + human_effect
5	700	7	3	21	0.672	0.992	0.724	S_Order + Runoff_9 + Runoff_12 + Runoff_8 + Runoff_10 + Runoff_7 + Bio_temp + Runoff_11 + SoilWet + Runoff_5 + Runoff_1 + Runoff_4 + Mean_Slope + ETPot + ET + Area_SqKm + Runoff_2 + Runoff_3 + Latitude + BIOME_NAME + human_effect
6	300	10	3	20	0.672	0.995	0.721	Runoff_12 + Runoff_10 + Runoff_9 + Bio_temp + Runoff_7 + Runoff_8 + Runoff_11 + Runoff_1 + SoilWet + Runoff_5 + Mean_Slope + Area_SqKm + ETPot + ET + Runoff_4 + Runoff_2 + Runoff_3 + Latitude + BIOME_NAME + human_effect
7	300	10	3	19	0.671	0.994	0.722	Runoff_12 + Runoff_9 + Runoff_10 + Runoff_8 + Runoff_7 + Runoff_11 + SoilWet + Runoff_1 + Runoff_5 + Mean_Slope + Runoff_4 + Area_SqKm + ETPot + ET + Runoff_2 + Runoff_3 + Latitude + BIOME_NAME + human_effect
8	500	9	7	18	0.672	0.994	0.723	Runoff_9 + Runoff_12 + Runoff_10 + Runoff_11 + Runoff_8 + SoilWet + Runoff_1 + Runoff_5 + Runoff_4 + Mean_Slope + Area_SqKm + ET + ETPot + Runoff_2 + Runoff_3 + Latitude + BIOME_NAME + human_effect
9	700	11	3	17	0.671	0.998	0.719	Runoff_10 + Runoff_9 + Runoff_8 + Runoff_11 + SoilWet + Runoff_1 + Runoff_5 + Runoff_4 + Mean_Slope + Area_SqKm + ETPot + ET + Runoff_2 + Runoff_3 + Latitude + BIOME_NAME + human_effect
10	900	11	3	16	0.669	0.998	0.719	Runoff_9 + Runoff_11 + Runoff_8 + SoilWet + Runoff_5 + Runoff_1 + Runoff_4 + Mean_Slope + ETPot + ET + Area_SqKm + Runoff_2 + Runoff_3 + Latitude + BIOME_NAME + human_effect
11	300	9	1	15	0.668	0.996	0.721	Runoff_11 + Runoff_8 + SoilWet + Runoff_4 + Runoff_5 + Runoff_1 + Mean_Slope + ETPot + ET + Area_SqKm + Runoff_2 + Runoff_3 + Latitude + BIOME_NAME + human_effect
12	300	7	2	14	0.666	0.992	0.723	Runoff_11 + Runoff_8 + Runoff_1 + Runoff_5 + Runoff_4 + Mean_Slope + ETPot + ET + Area_SqKm + Runoff_2 + Runoff_3 + Latitude + BIOME_NAME + human_effect
13	700	5	2	13	0.663	0.990	0.725	Runoff_5 + Runoff_8 + Runoff_1 + Runoff_4 + Mean_Slope + Area_SqKm + ET + ETPot + Runoff_2 + Runoff_3 + Latitude + BIOME_NAME + human_effect
14	100	3	6	12	0.664	0.986	0.730	Runoff_1 + Runoff_5 + Area_SqKm + Runoff_4 + Mean_Slope + ET + ETPot + Runoff_2 + Runoff_3 + Latitude + BIOME_NAME + human_effect
15	300	4	2	11	0.666	0.997	0.722	Area_SqKm + Mean_Slope + ET + Runoff_4 + ETPot + Runoff_5 + Runoff_2 + Runoff_3 + Latitude + BIOME_NAME + human_effect
16	500	3	2	10	0.664	0.991	0.726	Area_SqKm + Mean_Slope + ET + ETPot + Runoff_4 + Runoff_2 + Runoff_3 + Latitude + BIOME_NAME + human_effect
**17**	**500**	**4**	**3**	**9**	**0.661**	**0.984**	**0.730**	**Mean_Slope + ET + ETPot + Runoff_4 + Runoff_2 + Runoff_3 + Latitude + BIOME_NAME + human_effect**
18	900	3	1	8	0.662	0.981	0.731	Runoff_4 + ET + ETPot + Runoff_2 + Runoff_3 + Latitude + BIOME_NAME + human_effect
19	500	3	3	7	0.673	0.990	0.726	Runoff_4 + ETPot + Runoff_2 + Runoff_3 + Latitude + BIOME_NAME + human_effect
20	300	2	1	6	0.663	0.971	0.738	ETPot + Runoff_2 + Runoff_3 + Latitude + BIOME_NAME + human_effect
21	100	2	1	5	0.684	1.000	0.721	Runoff_3 + Runoff_2 + Latitude + BIOME_NAME + human_effect
22	700	2	1	4	0.689	1.006	0.716	Runoff_3 + Latitude + BIOME_NAME + human_effect
23	500	1	2	3	0.710	1.008	0.715	Latitude + BIOME_NAME + human_effect

Supplementary Table 7 showing model performance in R. Performance metrics (in log space) of the random forest models are split into training and testing data for the final model to predict each nitrogen (NO_3-N = nitrate-nitrogen, TN = total nitrogen) and P (DRP = dissolved reactive phosphorus, TP = total phosphorus) fraction.

Analyte	Dataset	Mean absolute error	Root mean square error	R^2
DRP	Train	0.366	0.615	0.97
	Test	0.974	1.650	0.76
TP	Train	0.376	0.538	0.94
	Test	1.056	1.540	0.55
NNN	Train	0.307	0.423	0.96
	Test	0.906	1.253	0.71
TN	Train	0.384	0.557	0.96
	Test	0.972	1.502	0.74

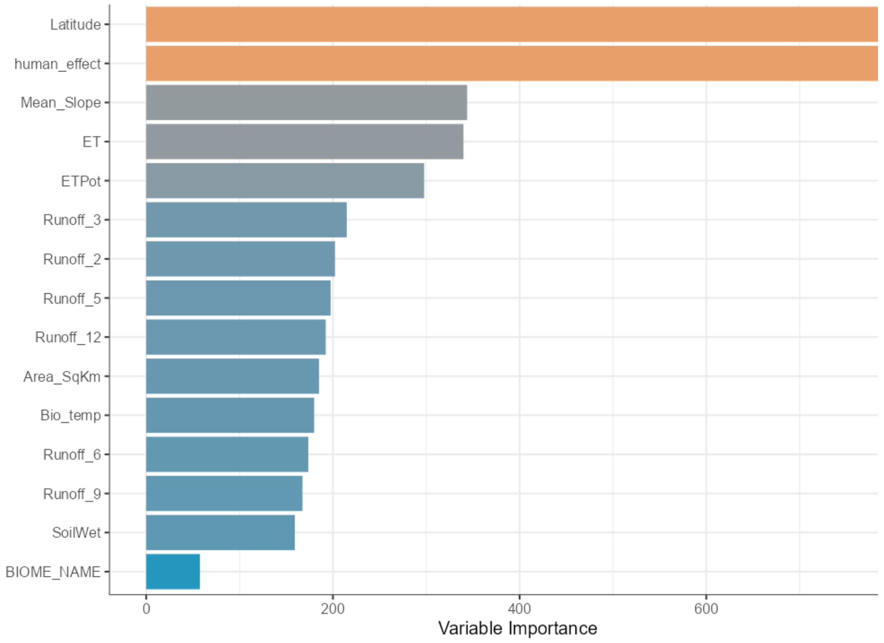

Supplementary Fig 3 showing the relative importance of predictor variables in the final model's estimation of dissolved reactive phosphorus concentration as implemented in R.

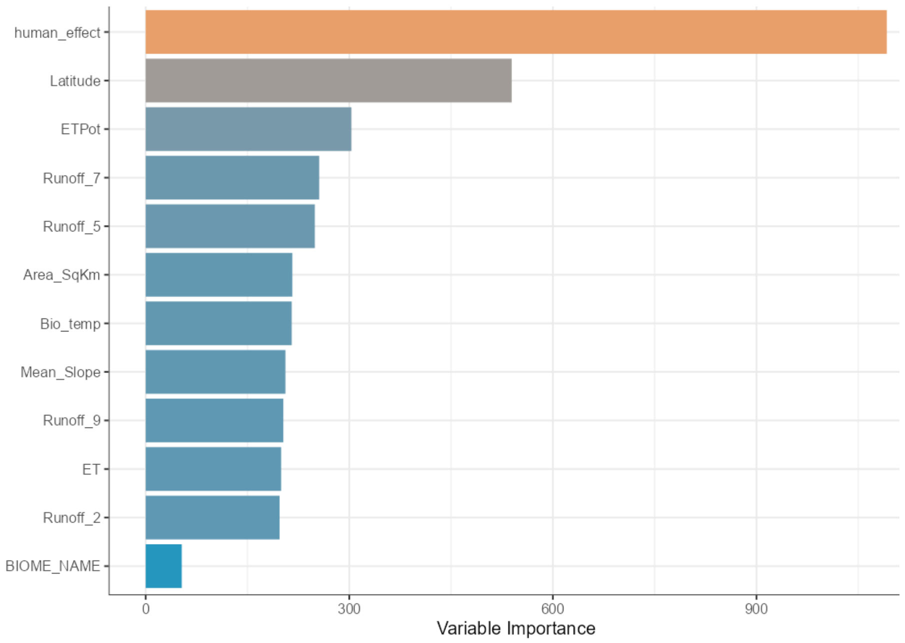

Supplementary Fig 4. showing the relative importance of predictor variables in the final model's estimation of total phosphorus concentration as implemented in R.

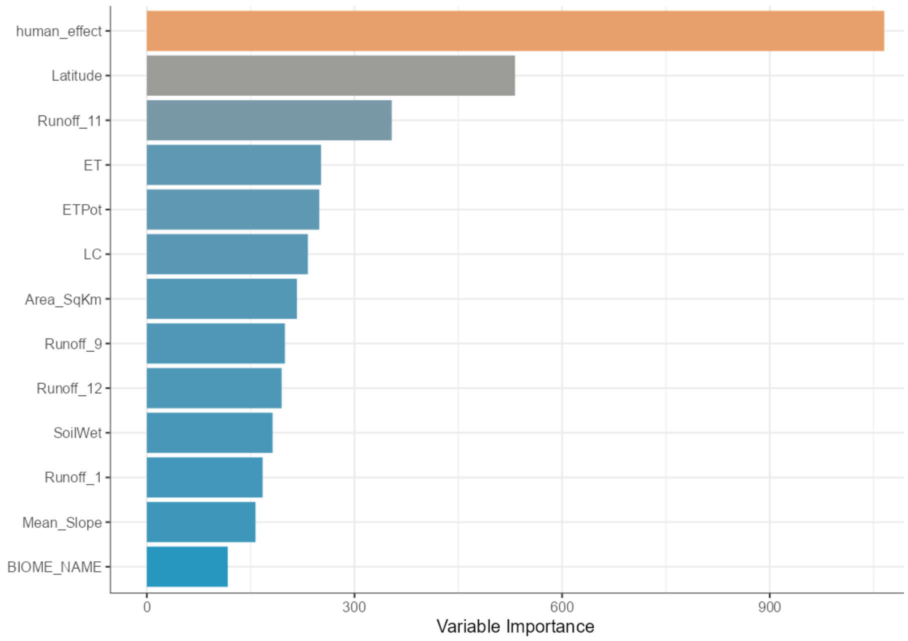

Supplementary Fig 5. showing the relative importance of predictor variables in the final model's estimation of nitrate-nitrogen concentration as implemented in R.

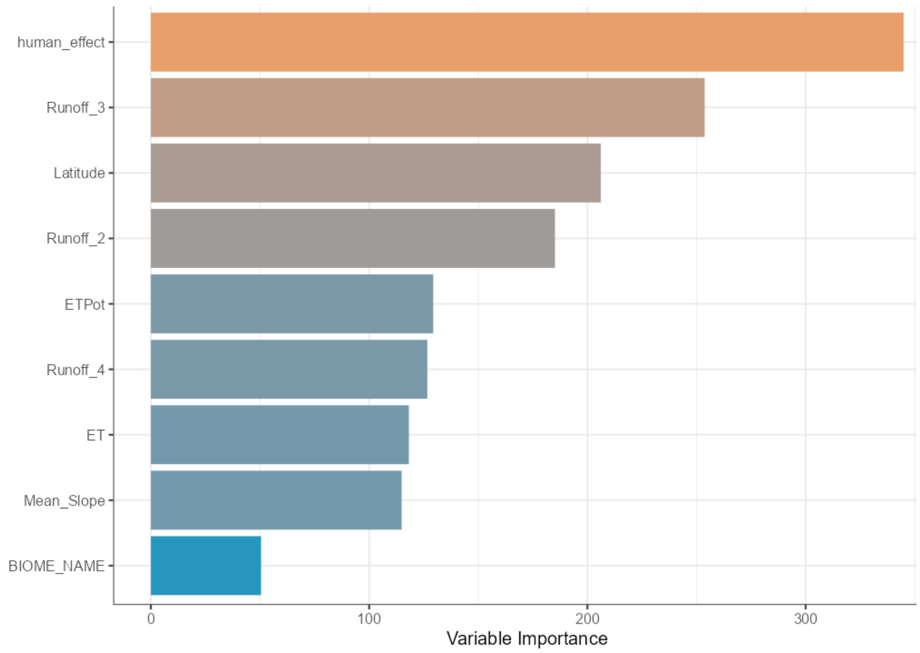

Supplementary Fig 6. showing the relative importance of predictor variables in the final model's estimation of total nitrogen concentration as implemented in R.

Supplementary Note 3: Implementation of the chosen model in Python

The performance of the chosen model (implemented in Python) is shown in Supplementary Table 8. The performance metrics, the coefficient of determination, mean absolute error and mean square error are acceptable [16]. Moreover they are better than previous attempts to model these analytes and derive likely effects on periphyton at a global [7] and national/regional scale [17]. Human effect was the most important predictor in each of the models followed by latitude and biophysical (e.g., mean slope) or climate conditions (e.g., runoff or potential evapotranspiration - ETPot). This order of effect is consistent with other models at the global [18], continental [19] or regional [20] scales. However, it is expected that at smaller scales, land use would become more important as biophysical or climate variation wanes [21]. Biomes were likely too large to capture farm scale changes in land use and the intensity of land management, both known to dominate catchment-scale losses [22].

Supplementary Table 8. showing model performance in Python. Performance metrics (in log space) of the random forest models are split into training and testing data for the final model to predict each nitrogen (NO_3-N = nitrate-nitrogen, TN = total nitrogen) and P (DRP = dissolved reactive phosphorus, TP = total phosphorus) fraction.

Analyte	Dataset	Mean absolute error	Root mean square error	R^2
DRP	Train	0.235	0.148	0.96
	Test	0.643	0.962	0.75
NO_3-N	Train	0.260	0.126	0.95
	Test	0.737	0.992	0.57
TP	Train	0.259	0.133	0.94
	Test	0.741	1.043	0.61
TN	Train	0.249	0.141	0.93
	Test	0.598	0.815	0.60

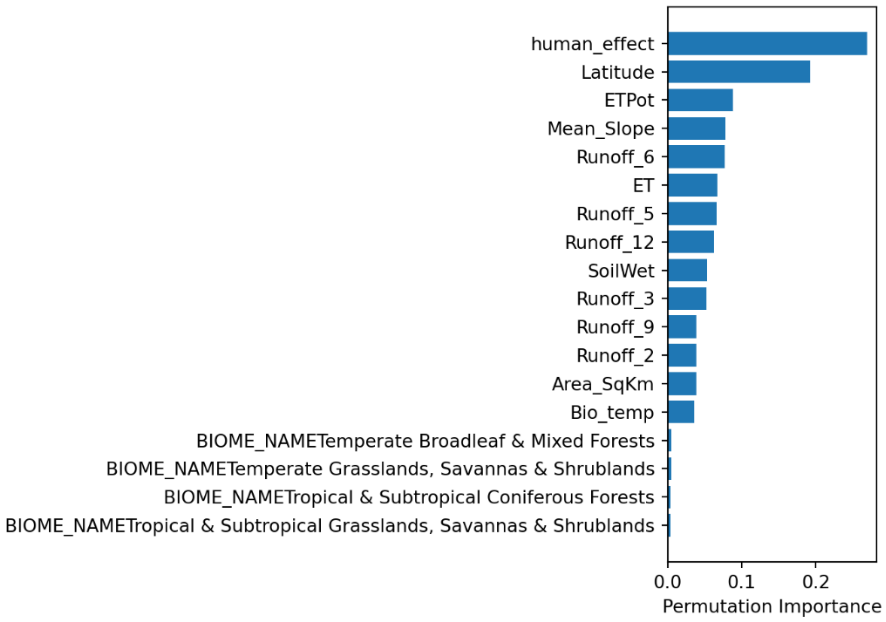

Supplementary Fig 7. showing the relative importance of predictor variables in the final model's estimation of dissolved reactive phosphorus concentration as implemented in Python.

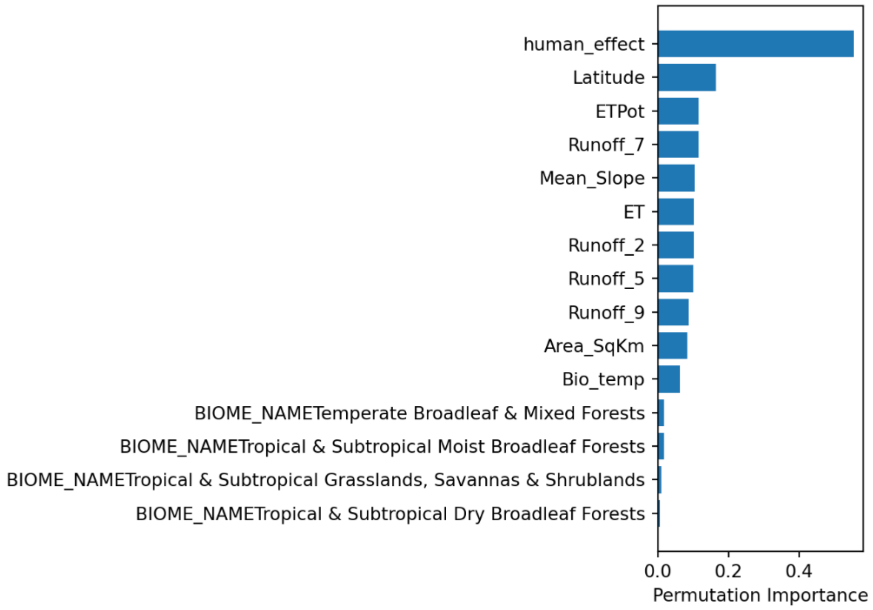

Supplementary Fig 8. showing the relative importance of predictor variables in the final model's estimation of total phosphorus concentration as implemented in Python.

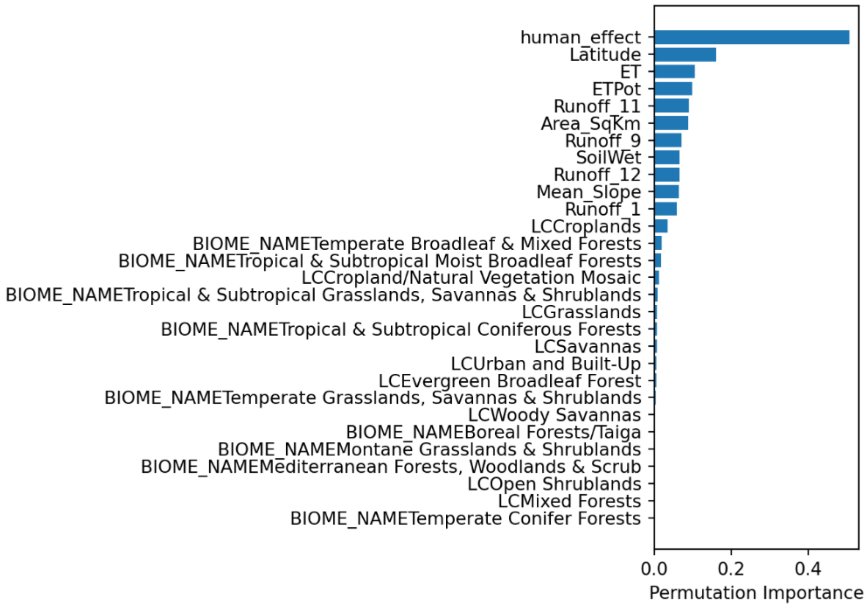

Supplementary Fig 9. showing the relative importance of predictor variables in the final model's estimation of nitrate-nitrogen concentration as implemented in Python.

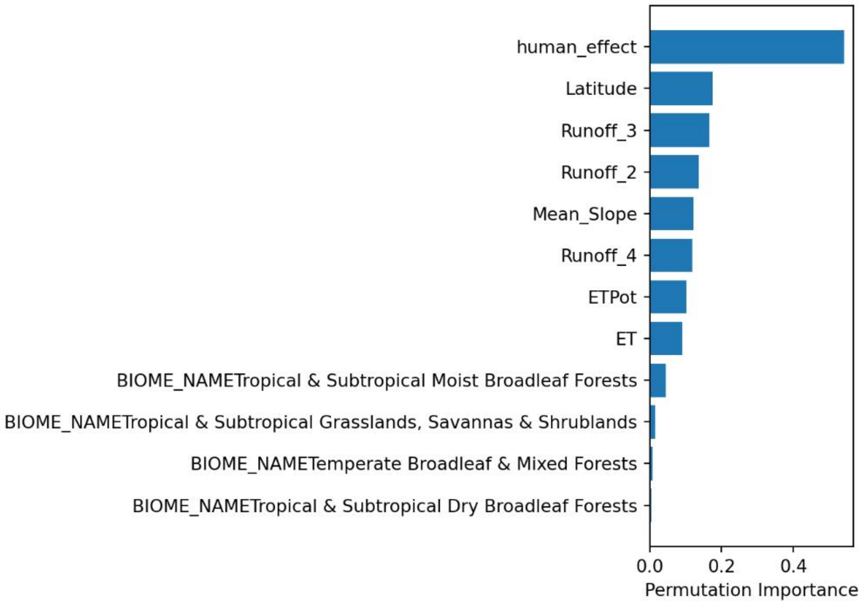

Supplementary Fig 10. showing the relative importance of predictor variables in the final model's estimation of total nitrogen concentration as implemented in Python.

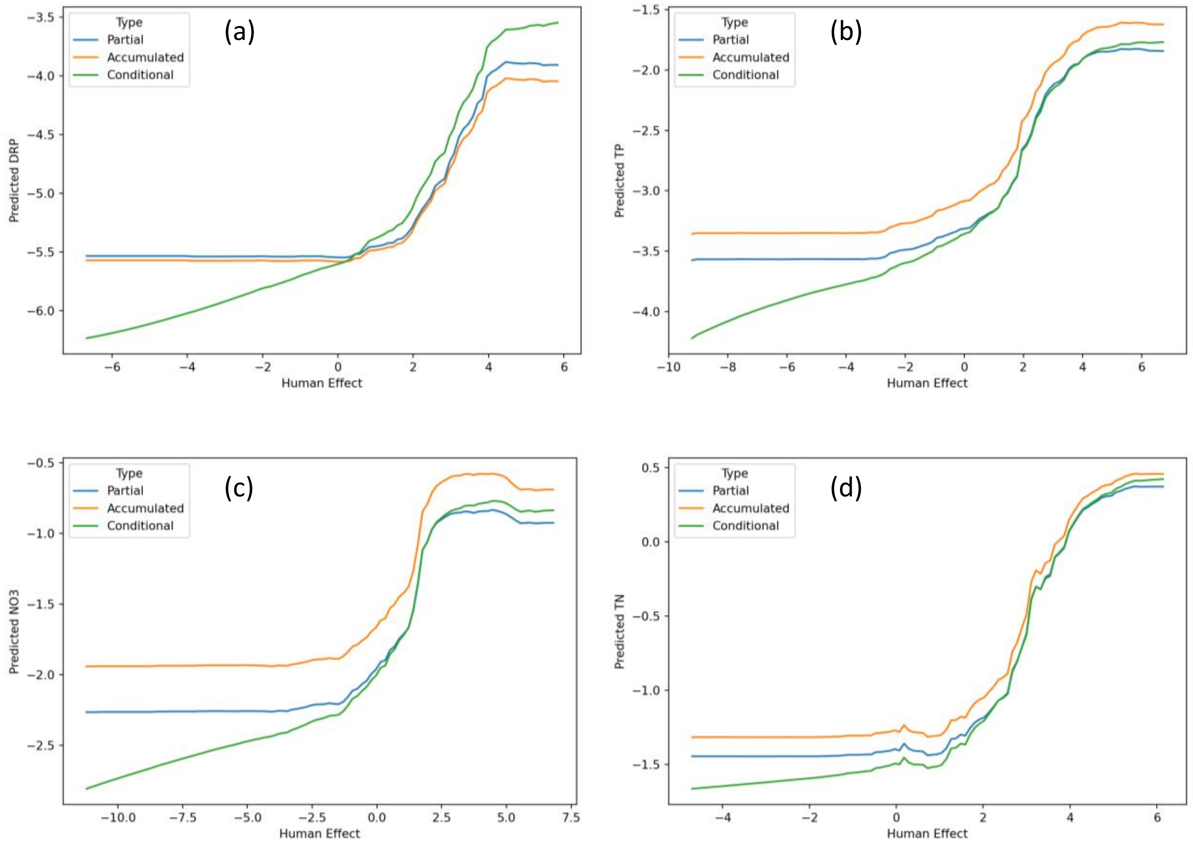

Supplementary Fig 11 showing partial dependence profile for the effect of Human Effect on each nitrogen and phosphorus fraction. These data are for a = DRP, dissolve reactive phosphorus; b = NO_3-N, nitrate-nitrogen; c = TP, total phosphorus; d = TN, total nitrogen concentrations as implemented in Python.

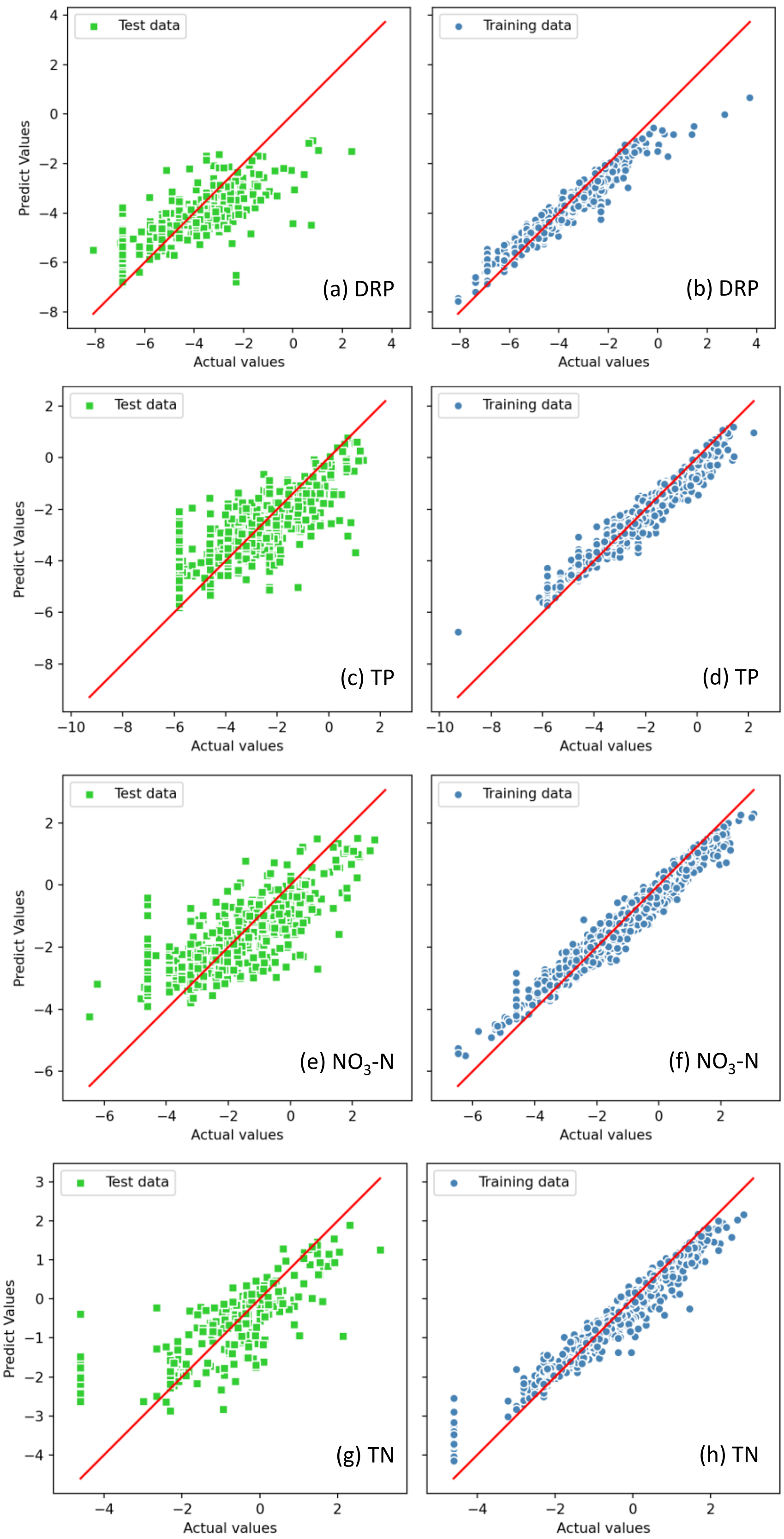

Supplementary Fig 12 showing plots in log space of predicted versus actual (viz observed data) for the training and test data as implemented in Python. The panels refer to a, b = DRP, dissolve reactive phosphorus; c, d = TP, total phosphorus; e, f = NO_3-N, nitrate-nitrogen; g, h = TN, total nitrogen. The red line is the 1:1 line.

Supplementary Note 4: Spatial performance and representativeness

We examined the spatial performance of each model by plotting the residuals as the proportional difference between the observed and predicted concentrations (Supplementary Fig 13). The median residual values ranged from 0.36 for NO_3-N to 0.52 for TN. Generally, residuals were evenly spread across the globe, except for a cluster of high residuals located in New Zealand commensurate with generally low observational concentrations. This cluster infers that a regional model may perform better.

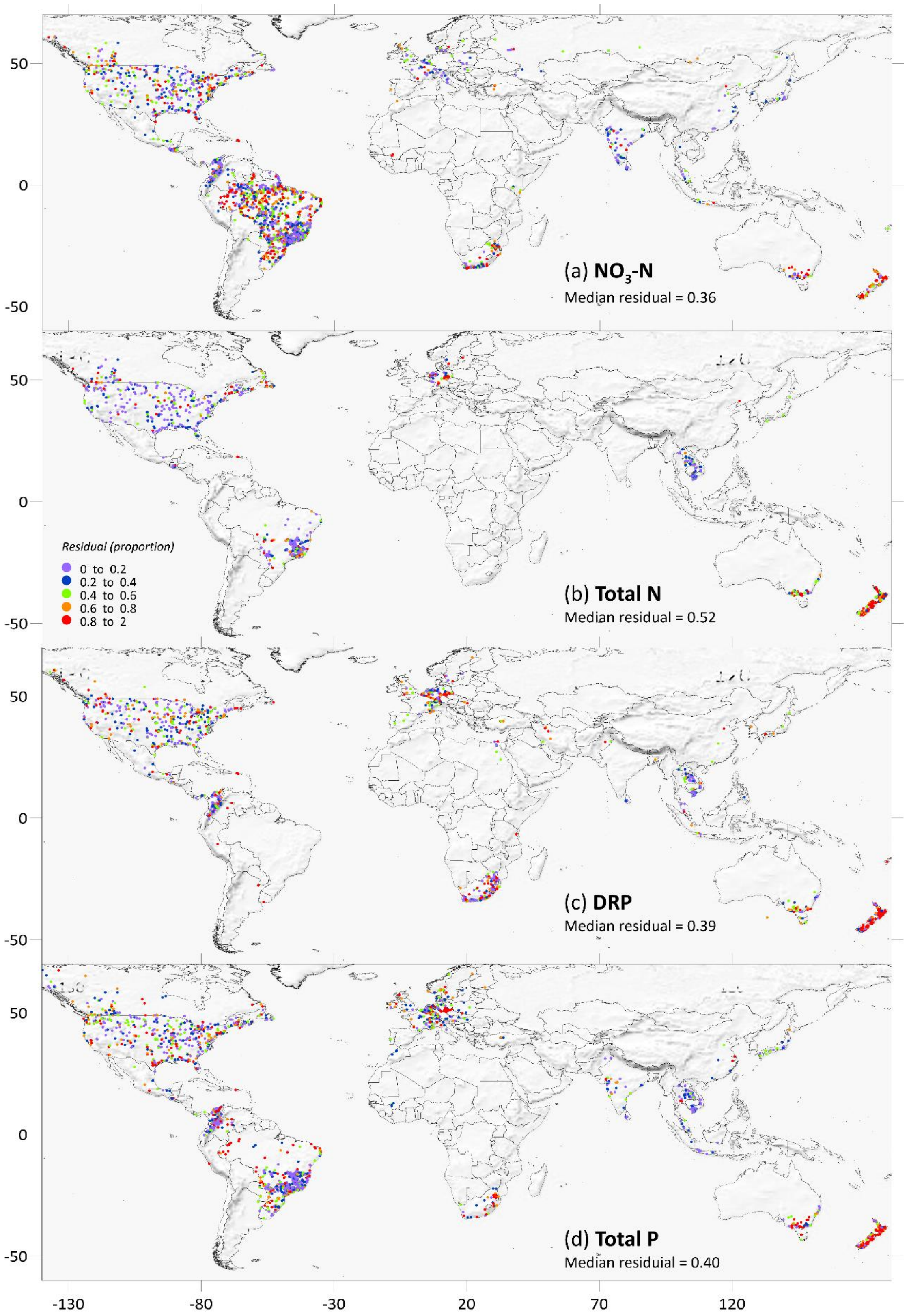

Supplementary Fig 13 showing the residuals for each site plotted as a proportion of the site concentration. (a) NO_3-N = nitrate-nitrogen; (b) TN = total nitrogen; (c) DRP = dissolve reactive phosphorus; (d) TP = total phosphorus;. Administrative boundaries from GADM (https://gadm.org/data.html).

Supplementary Note 5: Performance of the model in estimating reference conditions

Our approach makes several assumptions, *viz*. that: (1) our human effect variable is a good surrogate of anthropogenic influence on water quality analytes; (2) the number of sites used to fit the model are a representative, unbiased sample of the population of sites within a class; and (3) the estimate can be relied on and was not unduly influenced by other variables not included in the model.

As noted in Supplementary Note 1 and Supplementary Note 3, human effect was well characterised by variables thought likely to influence anthropogenic impact. Furthermore, the performance of the model to predict analyte concentrations was good and included human effect as the most important variable. Other studies have similarly shown the variables included in our human effect term like intensive agriculture (cropland or pasture) as prime drivers of reference conditions either as individual terms or grouped by biophysical and climatic classes [23,24]

In our analysis the success of the model is determined by the spread in the data, with an accurate estimate of reference conditions dependent on having sufficient data of low human effect to "anchor" the prediction. Out of 14241 catchments, 6128 had an Olsen P concentration ≤ 5 and 1577 had ≥ 80% forest cover. Some 615 (or 4.3%) met both conditions and had a low population density. Although the proportion of catchments with different concentrations of Olsen P, forest cover and population density vary across the database, we can say that the proportion was similar those with high Olsen P (>90th percentile; 42 mg kg^{-1}) and <20% forest cover [25]. This infers that the regression is equally leveraged by the proportion of sites with low and high human effect.

Finally, while we used many sites in our analysis, some biomes were underrepresented (e.g., Deserts and Xeric Shrublands and Borel Forests and Taiga). Although the model's performance suggests that estimates for reference conditions could be used to inform management, we chose to exclude these biomes from our continental calculations. This decision was also informed by the low likelihood that flowing streams and rivers would originate in these biomes (e.g., too dry, or too cold).

Even in areas where there are many sites, if there are no true reference sites (i.e., not just under minimally disturbed conditions), there is a possibility that reference conditions may not be well estimated due to the exclusion of natural factors. Such factors include, but are not limited to, climate and hydrologic variation [26]. Other studies have incorporated flow into regression models to help estimate nutrient concentrations under reference conditions [27]. Our choice of the median was deliberate to avoid undue bias by either low or high flow events. While this also means that values for some analytes will be different if flow dependant, and potentially under- or over-estimated at certain times of year, it avoids complexity when defining a value for a catchment or biome. Any variation due to flow would also be encompassed and expressed in the respective confidence intervals.

A final caveat is that our analysis did not account for trend over the period of record. However, the median trend for all analytes over the period of record in most developed countries is generally small (< 1% of site median values per annum [28,29]).

Supplemantary Note 6: Global reference values from the literature

Data (Supplementary Fig 14) were sourced from 37 global studies of reference conditons for nutrient concentrations established using least disturbed conditions represent a low (usually the 25th) percentile of concentrations for a representative set of sites, minimally disturbed conditions where sites are confirmed to have little human influence (e.g., <5% agricultural land), or statistical models that relate the level of human influence to nutrient concentrations and use this relationship to determine concentrations at zero human influence (i.e., the intercept of a regression relationship). The data were natural log-transformed and a general linear model fitted to either TN and TP (too few data were avilable for DRP or NO_3-N) with the main method of determining reference conditions and country nested within method as a fixed effect. For TN method and the country by method interaction were significant (P<0.001) with the model accounting for 54% of the variaiton in

concentration. For TP the country by method interacton was significant (but method not) with the the model accounting for 45% of the variation in concentrations. The respective regression equatons were:

LnTN = -0.590 + 0.443 TNmethod_LDC - 0.525 TNmethod_MDC + 0.082 TNmethod_Statistical - 0.254 TNCoutry(TNmethod)_Australia(LDC) + 0.695 TNCoutry(TNmethod)_China(LDC) - 0.442 TNCoutry(TNmethod)_United States(LDC) - 0.089 TNCoutry(TNmethod)_Brazil(MDC) + 0.140 TNCoutry(TNmethod)_Finland(MDC) - 0.265 TNCoutry(TNmethod)_Norway(MDC) + 0.013 TNCoutry(TNmethod)_Sweden(MDC) + 0.201 TNCoutry(TNmethod)_Venuzeuela(MDC) + 0.766 TNCoutry(TNmethod)_Brazil(Statistical) + 0.188 TNCoutry(TNmethod)_Canada(Statistical) + 1.096 TNCoutry(TNmethod)_China(Statistical) - 1.393 TNCoutry(TNmethod)_New Zealand(Statistical) - 0.808 TNCoutry(TNmethod)_Turkey(Statistical) + 0.151 TNCoutry(TNmethod)_United States(Statistical)

LnTP = -3.429 + 0.221 TPmethod_LDC - 0.238 TPmethod_MDC + 0.017 TPmethod_Statistical + 0.134 TPcountry(TPmethod)_Australia(LDC) + 0.307 TPcountry(TPmethod)_China(LDC) - 2.091 TPcountry(TPmethod)_Cyprus (LDC) + 0.212 TPcountry(TPmethod)_Cyprus, France, Italy, UK(LDC) + 1.598 TPcountry(TPmethod)_Germany(LDC) + 0.192 TPcountry(TPmethod)_Italy(LDC) - 0.353 TPcountry(TPmethod)_United States(LDC) + 0.170 TPcountry(TPmethod)_Brazil(MDC) - 0.245 TPcountry(TPmethod)_China(MDC) + 1.627 TPcountry(TPmethod)_Cote d'Ivoire(MDC) - 0.273 TPcountry(TPmethod)_Finland(MDC) - 0.960 TPcountry(TPmethod)_Norway(MDC) + 0.854 TPcountry(TPmethod)_Portugal(MDC) - 0.950 TPcountry(TPmethod)_Sweden(MDC) + 0.023 TPcountry(TPmethod)_United Kingdom(MDC) - 0.245 TPcountry(TPmethod)_Venuzeuela(MDC) + 1.792 TPcountry(TPmethod)_Brazil(Statistical) + 0.177 TPcountry(TPmethod)_Canada(Statistical) + 0.167 TPcountry(TPmethod)_China(Statistical) - 1.042 TPcountry(TPmethod)_New Zealand(Statistical) - 0.744 TPcountry(TPmethod)_Turkey(Statistical) - 0.351 TPcountry(TPmethod)_United States(Statistical)

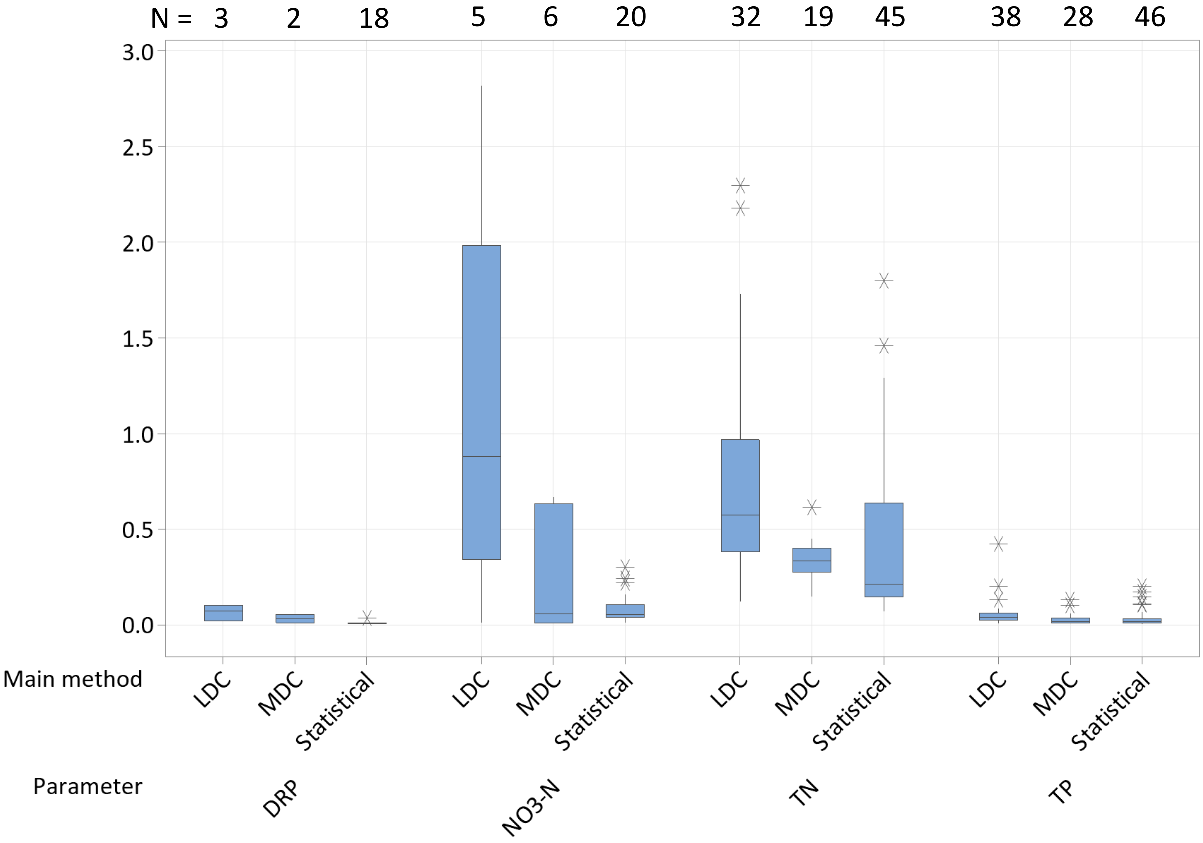

Supplementary Fig 14 showing box plots showing the concentration of nutrient reference conditions published in the literature (see Supplementary Table 9) by the main method of

determination. Box plots consist of the 5, 25, 50, 75, and 95th percentiles (and outliers) for least disturbed condition [LDC], minimally disturbed condition [MDC], or statistical methods. Also given are the count (N) for each method. A general linear model of the natural log-transformed concentrations of TN and TP indicated difference in reference conditions by method for TN but not for TP (DRP and NO_3-N had too few data to make meaningful contrasts).

Supplementary Table 9 showing examples of reference conditions by different methods for nutrient concentrations in different jurisdictions. Methods are classified as per Hawkins, Olson and Hill [67] and Stoddard, et al. [68] into those that have defined minimally disturbed conditions (MDC; e.g., a maximum population density), least disturbed conditions (LDC; usually a percentile [e.g., 25th] of nutrient concentrations for a group of biophysically similar sites), and statistical which includes mechanistic models for a few sites or techniques that group sites by typologies or ecopregions of similar biophysical characteristics and relate concentrations in those groups to a level of human activity (e.g., percentage catchment area in agriuclutrual land use), predicting the concentrations for zero human activity. Target refers to where the study has linked the nutrient criteria to thresholds in specific biological communities. If nothing is specified, values are for thresholds in ecological health – including periphyton.

Analyte	Country	River, Basin or Typology	Method	Analyte median or range (mg L^{-1})	Ref
DRP	United Kingdom	Axe River	LDC	0.02	30
DRP	Germany		LDC	0.1	31
DRP	Europe-14 member states		LDC	0.020-0.040	32
DRP	Mediterranean	Typology	LDC	0.07	33
DRP	Cote d'Ivoire		MDC	0.054	34
DRP	Greece		MDC	0.01	35
DRP	Spain		MDC	0.035-0.045	36
DRP	New Zealand	Cool dry lake	Statistical	0.003	24
DRP	New Zealand	Cool dry lowlands	Statistical	0.006	24
DRP	New Zealand	Cool dry mountainous	Statistical	0.004	24
DRP	New Zealand	Cool dry uplands	Statistical	0.003	24
DRP	New Zealand	Cool extremely wet lake	Statistical	0.002	24
DRP	New Zealand	Cool extremely wet lowlands	Statistical	0.007	24
DRP	New Zealand	Cool extremely wet mountainous	Statistical	0.003	24
DRP	New Zealand	Cool extremely wet uplands	Statistical	0.005	24
DRP	New Zealand	Cool wet lake	Statistical	0.002	24
DRP	New Zealand	Cool wet lowlands	Statistical	0.008	24
DRP	New Zealand	Cool wet mountainous	Statistical	0.003	24
DRP	New Zealand	Cool wet uplands	Statistical	0.006	24
DRP	New Zealand	Warm dry lowlands	Statistical	0.008	24
DRP	New Zealand	Warm extremely wet lowlands	Statistical	0.004	24

DRP	New Zealand	Warm extremely wet uplands	Statistical	0.008	[24]
DRP	New Zealand	Warm wet lake	Statistical	0.032	[24]
DRP	New Zealand	Warm wet lowlands	Statistical	0.009	[24]
DRP	New Zealand	Warm wet uplands	Statistical	0.008	[24]
NO3-N	Cyprus		LDC	0.01	[37]
NO3-N	Cyprus, France, Italy, UK		LDC	2.82	[38]
NO3-N	Italy		LDC	0.88	[39]
NO3-N	United Kingdom	Axe River	LDC	0.672	[30]
NO3-N	Europe-14 member states		LDC	2.000-6.000	[32]
NO3-N	Mediterranean	Typology	LDC	1.15	[33]
NO3-N	Brazil	Cerrado	MDC	0.004	[40]
NO3-N	Brazil	Cerrado	MDC	0.008	[41]
NO3-N	Cote d'Ivoire		MDC	0.62	[34]
NO3-N	Greece		MDC	0.1	[35]
NO3-N	Portugal		MDC	0.67	[42]
NO3-N	Spain		MDC	0.015	[36]
NO3-N	New Zealand	Cool dry lake	Statistical	0.036	[24]
NO3-N	New Zealand	Cool dry lowlands	Statistical	0.042	[24]
NO3-N	New Zealand	Cool dry mountainous	Statistical	0.027	[24]
NO3-N	New Zealand	Cool dry uplands	Statistical	0.014	[24]
NO3-N	New Zealand	Cool extremely wet lake	Statistical	0.05	[24]
NO3-N	New Zealand	Cool extremely wet lowlands	Statistical	0.072	[24]
NO3-N	New Zealand	Cool extremely wet mountainous	Statistical	0.046	[24]
NO3-N	New Zealand	Cool extremely wet uplands	Statistical	0.046	[24]
NO3-N	New Zealand	Cool wet lake	Statistical	0.01	[24]
NO3-N	New Zealand	Cool wet lowlands	Statistical	0.115	[24]
NO3-N	New Zealand	Cool wet mountainous	Statistical	0.025	[24]
NO3-N	New Zealand	Cool wet uplands	Statistical	0.061	[24]
NO3-N	New Zealand	Warm dry lowlands	Statistical	0.157	[24]
NO3-N	New Zealand	Warm extremely wet lowlands	Statistical	0.063	[24]

NO3-N	New Zealand	Warm extremely wet uplands	Statistical	0.053	[24]
NO3-N	New Zealand	Warm wet lake	Statistical	0.219	[24]
NO3-N	New Zealand	Warm wet lowlands	Statistical	0.039	[24]
NO3-N	New Zealand	Warm wet uplands	Statistical	0.067	[24]
NO3-N	United States	New York	Statistical	0.3	[43]
NO3-N	United States	New York	Statistical	0.24	[44]
TN	Australia	Victoria	LDC	0.251	[45]
TN	Australia	Victoria	LDC	0.49	[45]
TN	Australia	Victoria	LDC	0.406	[45]
TN	Australia	Victoria	LDC	0.33	[45]
TN	Australia	Victoria	LDC	0.568	[45]
TN	Australia	Victoria	LDC	0.69	[45]
TN	Australia	Victoria	LDC	0.957	[45]
TN	Australia	Victoria	LDC	0.97	[45]
TN	Australia	Victoria	LDC	1.27	[45]
TN	Australia	Victoria	LDC	1.306	[45]
TN	Australia	Victoria	LDC	1.22	[45]
TN	China	Cao-E River	LDC	1.73	[46]
TN	United States	Ecoregion I	LDC	0.31	[47]
TN	United States	Ecoregion II	LDC	0.12	[47]
TN	United States	Ecoregion III	LDC	0.38	[47]
TN	United States	Ecoregion IV	LDC	0.56	[47]
TN	United States	Ecoregion IX	LDC	0.69	[47]
TN	United States	Ecoregion IX	LDC	2.3	[48]
TN	United States	Ecoregion IX	LDC	0.53	[49]
TN	United States	Ecoregion V	LDC	0.88	[47]
TN	United States	Ecoregion VI	LDC	2.18	[47]
TN	United States	Ecoregion VII	LDC	0.54	[47]
TN	United States	Ecoregion VII	LDC	0.47	[48]
TN	United States	Ecoregion VIII	LDC	0.38	[47]

TN	United States	Ecoregion VIII	LDC	0.27	48
TN	United States	Ecoregion X	LDC	0.76	47
TN	United States	Ecoregion XI	LDC	0.31	47
TN	United States	Ecoregion XI	LDC	0.21	49
TN	United States	Ecoregion XI	LDC	0.58	48
TN	United States	Ecoregion XII	LDC	0.9	47
TN	United States	Ecoregion XIV	LDC	0.71	47
TN	United States	Ecoregion XIV	LDC	1.26	48
TN	Brazil	Sao Paulo	MDC	0.3	50
TN	Venezuela	Caura River	MDC	0.401	51
TN	Europe	Typology	MDC	0.250-35.0	52
TN	Finland	RN1	MDC	0.335	53
TN	Finland	RN3	MDC	0.45	53
TN	Finland	RN4	MDC	0.335	53
TN	Finland	RN5	MDC	0.335	53
TN	Finland	RN9	MDC	0.45	53
TN	Norway	Clay rivers	MDC	0.325	53
TN	Norway	RN1	MDC	0.275	53
TN	Norway	RN3	MDC	0.275	53
TN	Norway	RN4	MDC	0.275	53
TN	Norway	RN5	MDC	0.15	53
TN	Norway	RN9	MDC	0.25	53
TN	Sweden	Clay rivers	MDC	0.613	53
TN	Sweden	RN1	MDC	0.306	53
TN	Sweden	RN3	MDC	0.424	53
TN	Sweden	RN4	MDC	0.337	53
TN	Sweden	RN5	MDC	0.147	53
TN	Sweden	RN9	MDC	0.341	53
TN	Brazil	Rio de Janeiro	Statistical	1.294	54
TN	Cambodia	Mekong River	Statistical	0.266-0.313	55

TN	Canada AB	Prairies	Statistical	0.98	[56]
TN	Canada BC	Montane Cordillera	Statistical	0.21	[56]
TN	Canada MB	Prairies and transition to Boreal Plans	Statistical	0.39	[56]
TN	Canada NB	Atlantic Maritime	Statistical	0.87	[56]
TN	Canada ON	Mixedwood Plains	Statistical	1.06	[56]
TN	Canada PEI	Atlantic Maritime	Statistical	1.21	[56]
TN	Canada QC	Mixedwood Plains	Statistical	1.19	[56]
TN	China	Haihe River	Statistical	1.8	[57]
TN	China	Luan River	Statistical	1.297-1.642	[58]
TN	China	Qing River	Statistical	0.724-1.288	[59]
TN	New Zealand	Cool dry lake	Statistical	0.14	[24]
TN	New Zealand	Cool dry lowlands	Statistical	0.207	[24]
TN	New Zealand	Cool dry mountainous	Statistical	0.115	[24]
TN	New Zealand	Cool dry uplands	Statistical	0.105	[24]
TN	New Zealand	Cool extremely wet lake	Statistical	0.142	[24]
TN	New Zealand	Cool extremely wet lowlands	Statistical	0.154	[24]
TN	New Zealand	Cool extremely wet mountainous	Statistical	0.084	[24]
TN	New Zealand	Cool extremely wet uplands	Statistical	0.104	[24]
TN	New Zealand	Cool wet lake	Statistical	0.08	[24]
TN	New Zealand	Cool wet lowlands	Statistical	0.272	[24]
TN	New Zealand	Cool wet mountainous	Statistical	0.074	[24]
TN	New Zealand	Cool wet uplands	Statistical	0.165	[24]
TN	New Zealand	Warm dry lowlands	Statistical	0.371	[24]
TN	New Zealand	Warm extremely wet lowlands	Statistical	0.164	[24]
TN	New Zealand	Warm extremely wet uplands	Statistical	0.15	[24]
TN	New Zealand	Warm wet lake	Statistical	0.26	[24]
TN	New Zealand	Warm wet lowlands	Statistical	0.178	[24]
TN	New Zealand	Warm wet uplands	Statistical	0.185	[24]
TN	Turkey	R1	Statistical	0.71	[60]
TN	Turkey	R10	Statistical	0.28	[60]

TN	Turkey	R11	Statistical	0.3	60
TN	Turkey	R12	Statistical	0.58	60
TN	Turkey	R13	Statistical	0.16	60
TN	Turkey	R14	Statistical	0.15	60
TN	Turkey	R15	Statistical	0.26	60
TN	Turkey	R16	Statistical	1.46	60
TN	Turkey	R17	Statistical	0.21	60
TN	Turkey	R2	Statistical	0.14	60
TN	Turkey	R3	Statistical	0.19	60
TN	Turkey	R4	Statistical	0.69	60
TN	Turkey	R5	Statistical	0.35	60
TN	Turkey	R6	Statistical	0.22	60
TN	Turkey	R7	Statistical	0.27	60
TN	Turkey	R8	Statistical	0.07	60
TN	Turkey	R9	Statistical	0.11	60
TN	United States	New York	Statistical	0.7	43
TN	United States	14 Ecoregions	Statistical	0.020-2.300	47,49,61
TP	Australia	Victoria	LDC	0.024	45
TP	Australia	Victoria	LDC	0.024	45
TP	Australia	Victoria	LDC	0.025	45
TP	Australia	Victoria	LDC	0.024	45
TP	Australia	Victoria	LDC	0.019	45
TP	Australia	Victoria	LDC	0.035	45
TP	Australia	Victoria	LDC	0.054	45
TP	Australia	Victoria	LDC	0.039	45
TP	Australia	Victoria	LDC	0.086	45
TP	Australia	Victoria	LDC	0.063	45
TP	Australia	Victoria	LDC	0.067	45
TP	Australia	Victoria	LDC	0.06	45
TP	Australia	Victoria	LDC	0.42	45

TP	China	Cao-E River	LDC	0.055	46
TP	Cyprus		LDC	0.005	37
TP	Cyprus, France, Italy, UK		LDC	0.05	38
TP	Italy		LDC	0.049	39
TP	United States	Ecoregion I	LDC	0.047	47
TP	United States	Ecoregion II	LDC	0.01	47
TP	United States	Ecoregion III	LDC	0.022	47
TP	United States	Ecoregion IV	LDC	0.023	47
TP	United States	Ecoregion IX	LDC	0.037	47
TP	United States	Ecoregion IX	LDC	0.035	48
TP	United States	Ecoregion IX	LDC	0.06	49
TP	United States	Ecoregion V	LDC	0.067	47
TP	United States	Ecoregion VI	LDC	0.076	47
TP	United States	Ecoregion VII	LDC	0.033	47
TP	United States	Ecoregion VII	LDC	0.019	48
TP	United States	Ecoregion VIII	LDC	0.01	47
TP	United States	Ecoregion VIII	LDC	0.01	48
TP	United States	Ecoregion X	LDC	0.128	47
TP	United States	Ecoregion XI	LDC	0.01	47
TP	United States	Ecoregion XI	LDC	0.012	48
TP	United States	Ecoregion XI	LDC	0.02	49
TP	United States	Ecoregion XII	LDC	0.04	47
TP	United States	Ecoregion XIV	LDC	0.031	47
TP	United States	Ecoregion XIV	LDC	0.053	48
TP	Germany		LDC	0.2	31
TP	Brazil	Sao Paulo	MDC	0.006	62
TP	Brazil	Sao Paulo	MDC	0.04	50
TP	Brazil	Cerrado	MDC	0.035	40
TP	Brazil	Cerrado	MDC	0.1	41
TP	China	Tuojiang River	MDC	0.02	63

TP	Cote d'Ivoire		MDC	0.13	[34]
TP	Portugal	Mondego Basin	MDC	0.06	[42]
TP	United Kingdom	Lowland rivers (high alkalinity)	MDC	0.036	[64]
TP	United Kingdom	Lowland rivers (low alkalinity)	MDC	0.019	[64]
TP	Venezuela	Caura River	MDC	0.02	[51]
TP	Europe	Typology	MDC	0.008-0.660	[52]
TP	Finland	Clay rivers	MDC	0.04	[53]
TP	Finland	RN1	MDC	0.015	[53]
TP	Finland	RN3	MDC	0.02	[53]
TP	Finland	RN4	MDC	0.015	[53]
TP	Finland	RN5	MDC	0.015	[53]
TP	Finland	RN9	MDC	0.02	[53]
TP	Norway	Clay rivers	MDC	0.03	[53]
TP	Norway	RN1	MDC	0.009	[53]
TP	Norway	RN3	MDC	0.009	[53]
TP	Norway	RN4	MDC	0.009	[53]
TP	Norway	RN5	MDC	0.005	[53]
TP	Norway	RN9	MDC	0.008	[53]
TP	Sweden	Clay rivers	MDC	0.024	[53]
TP	Sweden	RN1	MDC	0.01	[53]
TP	Sweden	RN3	MDC	0.012	[53]
TP	Sweden	RN4	MDC	0.009	[53]
TP	Sweden	RN5	MDC	0.004	[53]
TP	Sweden	RN9	MDC	0.009	[53]
TP	Brazil	Rio de Janeiro	Statistical	0.198	[54]
TP	Cambodia	Mekong River	Statistical	0.025-0.039	[55]
TP	Canada AB	Prairies	Statistical	0.106	[56]
TP	Canada BC	Montane Cordillera	Statistical	0.02	[56]
TP	Canada MB	Prairies and transition to Boreal Plans	Statistical	0.102	[56]
TP	Canada NB	Atlantic Maritime	Statistical	0.013	[56]

TP	Canada ON	Mixedwood Plains	Statistical	0.026	[56]
TP	Canada PEI	Atlantic Maritime	Statistical	0.048	[56]
TP	Canada QC	Mixedwood Plains	Statistical	0.042	[56]
TP	China	Haihe River	Statistical	0.039	[57]
TP	China	Luan River	Statistical	0.041-0.049	[58]
TP	China	Qing River	Statistical	0.024-0.046	[59]
TP	New Zealand	Cool dry lake	Statistical	0.012	[24]
TP	New Zealand	Cool dry lowlands	Statistical	0.015	[24]
TP	New Zealand	Cool dry mountainous	Statistical	0.012	[24]
TP	New Zealand	Cool dry uplands	Statistical	0.008	[24]
TP	New Zealand	Cool extremely wet lake	Statistical	0.006	[24]
TP	New Zealand	Cool extremely wet lowlands	Statistical	0.013	[24]
TP	New Zealand	Cool extremely wet mountainous	Statistical	0.006	[24]
TP	New Zealand	Cool extremely wet uplands	Statistical	0.009	[24]
TP	New Zealand	Cool wet lake	Statistical	0.009	[24]
TP	New Zealand	Cool wet lowlands	Statistical	0.018	[24]
TP	New Zealand	Cool wet mountainous	Statistical	0.009	[24]
TP	New Zealand	Cool wet uplands	Statistical	0.01	[24]
TP	New Zealand	typology	Statistical	0.006-0.033	[24]
TP	New Zealand	Warm dry lowlands	Statistical	0.02	[24]
TP	New Zealand	Warm extremely wet lowlands	Statistical	0.008	[24]
TP	New Zealand	Warm extremely wet uplands	Statistical	0.012	[24]
TP	New Zealand	Warm wet lake	Statistical	0.033	[24]
TP	New Zealand	Warm wet lowlands	Statistical	0.019	[24]
TP	New Zealand	Warm wet uplands	Statistical	0.012	[24]
TP	United States	Typology	Statistical	0.006-0.080	[65]
TP	Turkey	R1	Statistical	0.017	[60]
TP	Turkey	R10	Statistical	0.008	[60]
TP	Turkey	R11	Statistical	0.008	[60]
TP	Turkey	R12	Statistical	0.008	[60]

TP	Turkey	R13	Statistical	0.008	[60]
TP	Turkey	R14	Statistical	0.065	[60]
TP	Turkey	R15	Statistical	0.142	[60]
TP	Turkey	R16	Statistical	0.003	[60]
TP	Turkey	R17	Statistical	0.032	[60]
TP	Turkey	R2	Statistical	0.007	[60]
TP	Turkey	R3	Statistical	0.023	[60]
TP	Turkey	R4	Statistical	0.169	[60]
TP	Turkey	R5	Statistical	0.018	[60]
TP	Turkey	R6	Statistical	0.031	[60]
TP	Turkey	R7	Statistical	0.005	[60]
TP	Turkey	R8	Statistical	0.003	[60]
TP	Turkey	R9	Statistical	0.015	[60]
TP	United States	New York	Statistical	0.03	[43]
TP	United States	New York	Statistical	0.018	[44]
TP	United States	14 Ecoregions	Statistical	0.006-0.080	[47,49,66]

Supplementary Note 7: Continental enrichment for each analyte by biome

Following the assessment of reference and current conditions for each nitrogen and phosphorus fraction, we calculated the enrichment as the difference between estimated current and estimated reference concentrations relative to reference conditions. Enrichment refers to the effect of human activity on the concentrations of nitrogen and phosphorus fractions for the period of record. These enrichment data are separated in Supplementary Table 10 into biomes for each continent.

Supplementary Table 10 giving the count and the median continential percentage enrichment for the median concentraitons of each analyte by biome. Data are given for all catchments and only those catchments exhibiting enrichment, when compared to the median concentrations for reference conditions.

Continent / biome	------ Dissolved reactive phosphorus ------				------------ Total phosphorus ------------				------------ Nitrate nitrogen ------------				------------ Total nitrogen ------------			
	All catchments		Catchments enriched >0%		All catchments		Catchments enriched >0%		All catchments		Catchments enriched >0%		All catchments		Catchments enriched >0%	
	Count	Median % enrichment	Count	Median % enrichment	Count	Median % enrichment	Count	Median % enrichment	Count	Median % enrichment	Count	Median % enrichment	Count	Median % enrichment	Count	Median % enrichment
**Africa**																
Deserts & Xeric Shrublands	1010	0.0	288	3.1	1010	0.0	222	1.8	1010	0.0	179	0.5	1010	0.0	267	4.7
Flooded Grasslands & Savannas	77	0.5	43	4.0	77	0.9	47	4.0	77	0.3	48	1.1	77	0.1	40	3.4
Mangroves	17	6.4	13	7.4	17	0.8	11	2.9	17	8.7	14	12.7	17	19.0	15	19.7
Mediterranean Forests, Woodlands & Scrub	125	12.5	98	29.0	125	11.0	95	19.0	125	6.4	87	22.2	125	9.1	86	18.5
Montane Grasslands & Shrublands	69	14.4	59	24.9	69	8.5	49	16.5	69	16.4	60	22.0	69	50.0	65	52.0
Temperate Conifer Forests	1	53.4	1	53.4	1	108.1	1	108.1	1	18.0	1	18.0	1	221.8	1	221.8
Tropical & Subtropical Dry Broadleaf Forests	24	6.7	18	10.7	24	2.9	17	5.4	24	1.5	18	3.2	24	0.0	3	40.4
Tropical & Subtropical Grasslands, Savannas & Shrublands	1856	3.2	1297	9.9	1856	1.6	1204	5.1	1856	1.1	1267	4.1	1856	5.1	1199	34.1
Tropical & Subtropical Moist Broadleaf Forests	373	18.3	313	28.3	373	8.7	317	10.9	373	9.2	329	12.2	373	42.6	295	66.5
**Asia**																
Boreal Forests/Taiga	499	0.0	114	0.8	499	0.0	207	1.0	499	0.0	250	0.5	499	1.8	408	2.5
Deserts & Xeric Shrublands	1341	0.0	426	2.0	1341	0.0	485	1.8	1341	0.0	405	1.0	1341	0.0	619	4.9
Flooded Grasslands & Savannas	42	1.2	28	3.7	42	2.1	34	2.7	42	0.3	30	2.4	42	1.8	31	3.2
Mangroves	20	8.0	18	8.5	20	5.7	17	7.4	20	3.4	17	3.9	20	10.1	13	14.0
Mediterranean Forests, Woodlands & Scrub	42	53.1	41	55.8	42	39.6	40	41.7	42	72.0	41	73.2	42	35.7	38	39.0
Montane Grasslands & Shrublands	228	0.2	126	2.1	228	1.0	152	2.1	228	1.5	170	3.0	228	9.0	202	16.1
Temperate Broadleaf & Mixed Forests	552	12.7	428	27.2	552	9.5	488	11.9	552	15.1	487	19.1	552	41.3	499	48.1
Temperate Conifer Forests	89	0.8	53	3.1	89	1.0	58	3.0	89	2.9	65	8.6	89	3.9	69	22.8
Temperate Grasslands, Savannas & Shrublands	416	0.0	141	6.8	416	1.8	323	3.0	416	0.6	253	6.1	416	22.6	398	28.2
Tropical & Subtropical Coniferous Forests	3	5.0	2	20.3	3	1.5	2	4.3	3	2.3	3	2.3	3	39.3	2	57.1
Tropical & Subtropical Dry Broadleaf Forests	160	31.1	153	32.8	160	0.0	60	7.5	160	11.7	129	22.6	160	23.6	139	27.2
Tropical & Subtropical Grasslands, Savannas & Shrublands	7	11.9	7	11.9	7	7.1	6	10.6	7	1.6	5	8.0	7	37.6	5	50.9
Tropical & Subtropical Moist Broadleaf Forests	867	19.5	753	25.6	867	6.4	675	9.8	867	13.1	818	14.1	867	26.6	674	50.3
Tundra	26	0.0	9	0.9	26	0.0	6	0.4	26	0.1	14	0.3	26	1.6	24	2.0
**Europe**																
Boreal Forests/Taiga	300	0.0	94	0.9	300	0.0	136	2.0	300	0.6	198	3.5	300	3.6	240	6.7
Deserts & Xeric Shrublands	24	0.1	12	2.0	24	0.4	13	2.0	24	0.0	9	2.3	24	0.4	15	2.7
Mediterranean Forests, Woodlands & Scrub	109	83.7	102	104.7	109	24.7	103	29.8	109	49.9	107	50.7	109	59.1	101	64.1
Temperate Broadleaf & Mixed Forests	554	6.1	324	54.4	554	8.2	525	10.8	554	38.8	506	44.8	554	78.8	551	79.2
Temperate Conifer Forests	28	41.1	25	47.6	28	32.6	28	32.6	28	64.0	28	64.0	28	136.6	28	136.6
Temperate Grasslands, Savannas & Shrublands	146	1.0	83	8.3	146	4.6	132	5.2	146	1.6	95	22.7	146	34.2	136	35.9
Tundra	54	0.0	24	1.3	54	0.0	14	1.5	54	0.2	32	1.4	54	2.0	47	2.2

**North America**																
Boreal Forests/Taiga	581	0.0	109	1.7	581	0.3	329	2.9	581	0.2	359	2.5	581	2.6	426	4.8
Deserts & Xeric Shrublands	309	0.0	115	2.4	309	0.0	141	2.0	309	0.0	119	0.9	309	0.0	150	4.9
Flooded Grasslands & Savannas	2	58.4	2	58.4	2	8.4	2	8.4	2	30.0	2	30.0	2	20.7	2	20.7
Mangroves	2	10.9	2	10.9	2	3.6	2	3.6	2	66.1	2	66.1	2	37.5	2	37.5
Mediterranean Forests, Woodlands & Scrub	21	5.6	16	13.9	21	2.0	16	11.2	21	7.2	17	7.7	21	1.4	13	13.5
Temperate Broadleaf & Mixed Forests	369	7.7	285	13.3	369	5.5	321	6.9	369	11.3	326	14.6	369	24.5	329	29.7
Temperate Conifer Forests	254	0.2	136	3.2	254	0.2	138	3.2	254	0.6	175	2.2	254	1.8	182	3.8
Temperate Grasslands, Savannas & Shrublands	393	1.6	238	11.2	393	4.0	314	6.0	393	1.1	251	8.5	393	26.6	335	41.5
Tropical & Subtropical Coniferous Forests	59	3.2	44	4.4	59	1.9	39	5.9	59	7.7	52	9.8	59	45.9	47	56.0
Tropical & Subtropical Dry Broadleaf Forests	71	6.3	61	11.6	71	0.5	39	5.4	71	21.8	60	28.4	71	37.5	63	39.2
Tropical & Subtropical Grasslands, Savannas & Shrublands	26	10.4	22	17.6	26	4.2	21	6.5	26	2.1	19	5.0	26	30.7	16	66.5
Tropical & Subtropical Moist Broadleaf Forests	89	18.8	83	23.9	89	11.0	79	12.0	89	19.2	87	20.9	89	70.3	81	72.6
Tundra	76	0.0	18	1.0	76	0.0	28	6.6	76	0.0	37	0.7	76	0.5	46	2.9
**Oceania**																
Deserts & Xeric Shrublands	421	0.0	20	0.9	421	0.0	22	1.4	421	0.0	17	0.3	421	0.0	28	2.8
Mediterranean Forests, Woodlands & Scrub	115	0.6	66	2.8	115	0.0	57	1.8	115	0.0	54	0.8	115	0.8	66	3.6
Montane Grasslands & Shrublands	4	1.1	3	2.1	4	1.3	2	3.2	4	0.0	1	42.0	4	13.4	3	26.1
Temperate Broadleaf & Mixed Forests	83	7.7	72	11.1	83	2.6	60	3.2	83	7.8	70	12.2	83	40.4	74	51.7
Temperate Grasslands, Savannas & Shrublands	80	0.0	20	2.6	80	0.0	26	1.4	80	0.0	21	0.5	80	0.0	32	4.3
Tropical & Subtropical Grasslands, Savannas & Shrublands	302	0.0	60	1.1	302	0.0	52	1.2	302	0.0	84	0.3	302	0.0	100	1.4
Tropical & Subtropical Moist Broadleaf Forests	12	10.6	11	11.9	12	8.2	9	11.0	12	7.1	11	7.4	12	0.0	4	0.9
**South America**																
Deserts & Xeric Shrublands	63	0.0	31	26.8	63	0.0	30	4.0	63	0.0	29	11.8	63	0.5	38	11.7
Flooded Grasslands & Savannas	27	0.0	5	0.9	27	0.0	12	2.8	27	0.0	14	1.3	27	0.0	11	1.8
Mangroves	16	4.6	15	4.6	16	8.4	16	8.4	16	6.3	16	6.3	16	9.8	12	21.6
Mediterranean Forests, Woodlands & Scrub	12	0.1	6	3.9	12	3.2	8	4.7	12	4.0	8	9.8	12	14.3	11	16.1
Montane Grasslands & Shrublands	63	0.0	23	1.7	63	0.1	33	0.6	63	0.4	43	1.0	63	0.9	51	1.1
Temperate Broadleaf & Mixed Forests	54	7.6	40	11.3	54	1.5	36	5.1	54	3.2	43	8.5	54	34.1	53	34.4
Temperate Grasslands, Savannas & Shrublands	218	2.0	173	3.4	218	0.8	136	2.0	218	0.9	152	2.2	218	2.3	160	4.8
Tropical & Subtropical Dry Broadleaf Forests	194	2.2	117	11.7	194	5.5	147	8.4	194	2.7	147	4.6	194	29.7	142	54.8
Tropical & Subtropical Grasslands, Savannas & Shrublands	455	0.0	208	3.8	455	1.4	265	5.6	455	0.8	309	3.3	455	0.0	209	3.9
Tropical & Subtropical Moist Broadleaf Forests	808	0.0	321	5.9	808	1.0	440	9.0	808	1.1	542	3.9	808	0.0	345	5.8

Supplementary Note 8: Representativeness of the outputs

Observations were available for 3679 out of 14241 catchments at level 6 of HydroBasins [69]. We tested if the count of sites across our observations was consistent with the count of catchments at level 6 of HydroBasins by comparing the proportion of catchments in each biome and a Chi-squared test. The output (Supplementary Table 11) indicated that there was a difference in the proportion of observations between biomes compared to the proportion at level 6 of HydroBasins caused largely by a paucity of observations in the Deserts & Xeric Shrublands and Boreal Forests/Taiga biomes (and an over-representation of observations in the Temperate Broadleaf & Mixed Forests biome). Owing to the poor representativeness and low likelihood of flowing water originating in the Deserts & Xeric Shrublands and Boreal Forests/Taiga biomes we chose to report continental statistics and present maps without predictions for these biomes.

Supplementary Table 11. Count of catchments (by biome) within the global HydroBasins (level 6) database and our observations along with the Chi-squared proportion indicating the leverage of each count biome in producing an expected count that is different (P<0.001) between HydroBasins and the observations.

Biome	Count HydroBasins L6	Count observations	Chi-sq test proportion
Boreal Forests/Taiga	1380	54	0.097
Deserts & Xeric Shrublands	3168	62	0.222
Flooded Grasslands & Savannas	148	16	0.010
Mangroves	55	9	0.004
Mediterranean Forests, Woodlands & Scrub	424	79	0.030
Montane Grasslands & Shrublands	364	56	0.026
Temperate Broadleaf & Mixed Forests	1613	559	0.113
Temperate Conifer Forests	372	107	0.026
Temperate Grasslands, Savannas & Shrublands	1253	163	0.088
Tropical & Subtropical Coniferous Forests	62	46	0.004
Tropical & Subtropical Dry Broadleaf Forests	449	192	0.032
Tropical & Subtropical Grasslands, Savannas & Shrublands	2646	670	0.186
Tropical & Subtropical Moist Broadleaf Forests	2151	1507	0.151
Tundra	156	12	0.011
Total	14241	3679	P value <0.001

Supplementary references

1 European Space Agency. *European Space Agency GlobCover Portal - GlobCover 2009*, <http://due.esrin.esa.int/page_globcover.php> (2010).

2 McDowell, R. W., Noble, A., Pletnyakov, P. & Mosley, L. Global database of riverine nitrogen and phosphorus loads and yields. https://doi.org/10.25400/lincolnuninz.11894697 (2020).

3 Center for International Earth Science Information Network - CIESIN - Columbia University & Centro Internacional de Agricultura Tropical - CIAT. Gridded Population of the World, Version 3 (GPWv3): Population Density Grid. (2005).

4 United States Department of the Interior - United States Geological Survey. HydroSHEDS. (U.S. Dept. of the Interior, U. S. Geological Survey,, Washinton D.C., 2008).

5 IUSS Working Group WRB. World Reference Base for Soil Resources 2014, update 2015 International soil classification system for naming soils and creating legends for maps. (Food and Agrculture Orgnisation, Rome, Italy, 2015).

6 World Bank. *GDP per capita (current US$)*, <https://data.worldbank.org/indicator/NY.GDP.PCAP.CD?view=map> (2018).

7 McDowell, R. W., Noble, A., Pletnyakov, P., Haggard, B. E. & Mosley, L. M. Global mapping of freshwater nutrient enrichment and periphyton growth potential. *Scientific Reports* **10**, 3568 (2020).

8 Willmott, C. J. & Matsuura, K. *Terrestrial Air Temperature and Precipitation: Monthly and Annual Time Series (1950 - 1999)*, <http://climate.geog.udel.edu/~climate/html_pages/download.html> (2001).

9 Fekete, B. M., Vörösmarty, C. J. & Grabs, W. *UNH / GRDC Composite Runoff Fields v1.0*, <http://www.compositerunoff.sr.unh.edu/> (2018).

10 Fick, S. E. & Hijmans, R. J. WorldClim 2: new 1-km spatial resolution climate surfaces for global land areas. *International Journal of Climatology* **37**, 4302-4315 (2017).

11 Dinerstein, E. *et al.* An Ecoregion-Based Approach to Protecting Half the Terrestrial Realm. *Bioscience* **67**, 534-545 (2017).

12 Sayre, R. *et al.* A New Map of Global Ecological Land Units - An Ecophysiographic Stratification Approach. 46 (Association of American Geographers, Washington D.C., 2014).

13 Abdi, H. Partial least squares regression and projection on latent structure regression (PLS Regression). *Wiley Interdisciplinary Reviews: Computational Statistics* **2**, 97-106 (2010).

14 McDowell, R. W., Rotz, C. A., Oenema, J. & Macintosh, K. A. Limiting grazing periods combined with proper housing can reduce nutrient losses from dairy systems. *Nature Food* **3**, 1065-1074 (2022).

15 Bowes, M. J., Smith, J. T., Jarvie, H. P. & Neal, C. Modelling of phosphorus inputs to rivers from diffuse and point sources. *Sci. Total Environ.* **395**, 125-138 (2008).

16 Moriasi, D. N., Arnold, J. G., Van Liew, M. W., Bingner, R. L., Harmel, R. D. & Veith, T. L. Model Evaluation Guidelines for Systematic Quantification of Accuracy in Watershed Simulations. *Transactions of the ASABE* **50**, 885-900 (2007).

17 Snelder, T. H., Moore, C. & Kilroy, C. Nutrient Concentration Targets to Achieve Periphyton Biomass Objectives Incorporating Uncertainties. *JAWRA Journal of the American Water Resources Association* **55**, 1443-1463 (2019).

18 Schulte-Uebbing, L. F., Beusen, A. H. W., Bouwman, A. F. & de Vries, W. From planetary to regional boundaries for agricultural nitrogen pollution. *Nature* **610**, 507-512 (2022).

19 Herlihy, A. T., Paulsen, S. G., Sickle, J. V., Stoddard, J. L., Hawkins, C. P. & Yuan, L. L. Striving for consistency in a national assessment: the challenges of applying a reference-condition approach at a continental scale. *J. N. Am. Benthol. Soc.* **27**, 860-877 (2008).

20 Chand, A. Nitrogen boundaries at regional scale. *Nature Food* **3**, 888-888 (2022).

21 McDowell, R. W., Cox, N. & Snelder, T. in *New Zealand Socity of Freshwater Sciences: Freshwaters and the Edge* (New Zealand Socity of Freshwater Sciences, Invercargill, New Zealand, 2016).
22 Oenema, J. & Oenema, O. Intensification of grassland-based dairy production and its impacts on land, nitrogen and phosphorus use efficiencies. *Front. Agr. Sci. Eng.* **8**, 130-147 (2021).
23 Dodds, W. K. & Oakes, R. M. A technique for establishing reference nutrient concentrations across watersheds affected by humans. *Limnol. Oceanogr. Methods* **2**, 333-341 (2004).
24 McDowell, R. W., Snelder, T. H., Cox, N., Booker, D. J. & Wilcock, R. J. Establishment of reference or baseline conditions of chemical indicators in New Zealand streams and rivers relative to present conditions. *Mar. Freshwat. Res.* **64**, 387-400 (2013).
25 McDowell, R. W., Pletnyakov, P., Noble, A. & Haygarth, P. M. A Global Database of Soil Plant Available Phosphorus. *figshare* https://doi.org/10.6084/m9.figshare.14241854 (2023).
26 Snelder, T. H., Larned, S. T., Fraser, C. & De Malmanche, S. Effect of climate variability on water quality trends in New Zealand rivers. *Mar. Freshwat. Res.* **73**, 20-34 (2022).
27 Suplee, M. W., Varghese, A. & Cleland, J. Developing Nutrient Criteria for Streams: An Evaluation of the Frequency Distribution Method1. *JAWRA Journal of the American Water Resources Association* **43**, 453-472 (2007).
28 European Environment Agency. *Waterbase - Water Quality*, <http://cmshare.eea.europa.eu/s/B9dGkQGHtJoqPqJ/download> (2020).
29 LAWA. Land, Air, Water, Aotearoa. (2015). < http://www.lawa.org.nz>.
30 Glavan, M., White, S. M. & Holman, I. P. Water quality targets and maintenance of valued landscape character – Experience in the Axe catchment, UK. *J. Environ. Manage.* **103**, 142-153 (2012).
31 Birk, S., van Kouwen, L. & Willby, N. Harmonising the bioassessment of large rivers in the absence of near-natural reference conditions – a case study of the Danube River. *Freshwat. Biol.* **57**, 1716-1732 (2012).
32 Pardo, I. *et al.* The European reference condition concept: A scientific and technical approach to identify minimally-impacted river ecosystems. *Sci. Total Environ.* **420**, 33-42 (2012).
33 Feio, M. J. *et al.* Least Disturbed Condition for European Mediterranean rivers. *Sci. Total Environ.* **476-477**, 745-756 (2014).
34 Kamelan, T. M., Diomande, A., Yao, K. M., Berte, S. & Koualelan, E. P. Reference values of the physico-chemical parameters of the water streams of Tai national Park (Côte d'Ivoire). *International Journal of Biological and Chemical Sciences* **16**, 1331-1349 (2022).
35 Skoulikidis, N. T., Amaxidis, Y., Bertahas, I., Laschou, S. & Gritzalis, K. Analysis of factors driving stream water composition and synthesis of management tools—A case study on small/medium Greek catchments. *Sci. Total Environ.* **362**, 205-241 (2006).
36 Sánchez-Montoya, M. D. M., Arce, M. I., Vidal-Abarca, M. R., Suárez, M. L., Prat, N. & Gómez, R. Establishing physico-chemical reference conditions in Mediterranean streams according to the European Water Framework Directive. *Water Res.* **46**, 2257-2269 (2012).
37 Cantonati, M. *et al.* Overwhelming role of hydrology-related variables and river types in driving diatom species distribution and community assemblage in streams in Cyprus. *Ecol. Indicators* **117**, 106690 (2020).
38 Erba, S. *et al.* The validation of common European class boundaries for river benthic macroinvertebrates to facilitate the intercalibration process of the Water Framework Directive. *Hydrobiologia* **633**, 17-31 (2009).
39 Erba, S., Buffagni, A., Cazzola, M. & Balestrini, R. Italian reference rivers under the Water Framework Directive umbrella: do natural factors actually depict the observed nutrient conditions? *Environmental Sciences Europe* **34**, 63 (2022).

40 Gucker, B., Boechat, I. G. & Giani, A. Impacts of agricultural land use on ecosystem structure and whole-stream metabolism of tropical Cerrado streams. *Freshwat. Biol.* **54**, 2069-2085 (2009).
41 Silva, J. S. O., da Cunha Bustamante, M. M., Markewitz, D., Krusche, A. V. & Ferreira, L. G. Effects of land cover on chemical characteristics of streams in the Cerrado region of Brazil. *Biogeochemistry* **105**, 75-88 (2011).
42 Chaves, M. L., Costa, J. L., Chainho, P., Costa, M. J. & Prat, N. Selection and validation of reference sites in small river basins. *Hydrobiologia* **573**, 133-154 (2006).
43 Smith, A. J. & Tran, C. P. A weight-of-evidence approach to define nutrient criteria protective of aquatic life in large rivers. *J. N. Am. Benthol. Soc.* **29**, 875-891 (2010).
44 Smith, A. J., Bode, R. W. & Kleppel, G. S. A nutrient biotic index (NBI) for use with benthic macroinvertebrate communities. *Ecol. Indicators* **7**, 371-386 (2007).
45 Newall, P. & Tiller, D. Derivation of Nutrient Guidelines for Streams in Victoria, Australia. *Environ. Monit. Assess.* **74**, 85-103 (2002).
46 Chen, J. & Lu, J. Establishment of reference conditions for nutrients in an intensive agricultural watershed, Eastern China. *Environmental Science and Pollution Research* **21**, 2496-2505 (2014).
47 USEPA. *Ecoregional Nutrient Criteria for Rivers and Streams (Website with a document for each Ecoregion of the US)*, <https://www.epa.gov/nutrient-policy-data/ecoregional-nutrient-criteria-rivers-and-streams> (2020).
48 Clune, J. W., Crawford, J. K. & Boyer, E. W. Nitrogen and Phosphorus Concentration Thresholds toward Establishing Water Quality Criteria for Pennsylvania, USA. *Water* **12**, 3550 (2020).
49 Longing, S. D. & Haggard, B. E. Distributions of Median Nutrient and Chlorophyll Concentrations across the Red River Basin, USA. *J. Environ. Qual.* **39**, 1966-1974 (2010).
50 Cunha, D. G. F., Dodds, W. K. & Carmo Calijuri, M. d. Defining Nutrient and Biochemical Oxygen Demand Baselines for Tropical Rivers and Streams in São Paulo State (Brazil): A Comparison Between Reference and Impacted Sites. *Environ. Manage.* **48**, 945-956 (2011).
51 Lewis Jr., W. M. Nitrogen and Phosphorus Runoff Losses from a Nutrient-Poor Tropical Moist Forest. *Ecology* **67**, 1275-1282 (1986).
52 Poikane, S. *et al.* Nutrient criteria for surface waters under the European Water Framework Directive: Current state-of-the-art, challenges and future outlook. *Sci. Total Environ.* **695**, 133888 (2019).
53 Skarbøvik, E. *et al.* Comparing nutrient reference concentrations in Nordic countries with focus on lowland rivers. *Ambio* **49**, 1771-1783 (2020).
54 Tromboni, F. & Dodds, W. K. Relationships Between Land Use and Stream Nutrient Concentrations in a Highly Urbanized Tropical Region of Brazil: Thresholds and Riparian Zones. *Environ. Manage.* **60**, 30-40 (2017).
55 Tromboni, F. *et al.* Changing Land Use and Population Density Are Degrading Water Quality in the Lower Mekong Basin. *Water* **13**, 1948 (2021).
56 Chambers, P. A., McGoldrick, D. J., Brua, R. B., Vis, C., Culp, J. M. & Benoy, G. A. Development of Environmental Thresholds for Nitrogen and Phosphorus in Streams. *J. Environ. Qual.* **41**, 7-20 (2012).
57 Wu, F., Fang, Y., Feng, M., Xie, Z., Zhu, L. & Feng, J. Developing Ecological Thresholds for Nitrogen and Phosphorus in the Haihe River Basin in China. *Int. J. Env. Res. Public Health* **19**, 16951 (2022).
58 Cheng, P. & Li, X. Establishing reference nutrient conditions using improved statistical methods in a river network with typical monsoon climatic pattern. *Ecol. Indicators* **89**, 260-268 (2018).
59 Chen, J., Li, F., Wang, Y. & Kong, Y. Estimating the nutrient thresholds of a typical tributary in the Liao River basin, Northeast China. *Scientific reports* **8**, 3810 (2018).

60 Ongun Sevindik, T., Çetin, T., Tunca, H., Güzel, U. & Tekbaba, A. G. Ecological status estimation of minimally disturbed rivers of the Akarçay Basin (Türkiye) using diatom indices. *Biologia* (2023).
61 Alexander, R. B., Johnes, P. J., Boyer, E. W. & Smith, R. A. A comparison of models for estimating the riverine export of nitrogen from large watersheds. *Biogeochemistry* **57**, 295-339 (2002).
62 Fonseca, B. M., de Mendonça-Galvão, L., Padovesi-Fonseca, C., de Abreu, L. M. & Fernandes, A. C. M. Nutrient baselines of Cerrado low-order streams: comparing natural and impacted sites in Central Brazil. *Environ. Monit. Assess.* **186**, 19-33 (2014).
63 Zhang, L., Wu, L., Wu, C., Jin, J., Huo, S. & Zhou, Y. Construction of lake reference conditions for nutrient criteria based on system dynamics modelling. *Ecol. Model.* **383**, 69-79 (2018).
64 UK Technical Advisory Group on the Water Framework Directive. Updated recommendations on phosphorus standards for rivers: River basin management (2015-2021). 13 (https://www.wfduk.org/sites/default/files/Media/UKTAG%20Phosphorus%20Standards%20for%20Rivers_Final%20130906_0.pdf, 2013).
65 Robertson, D. M., Saad, D. A. & Heisey, D. M. A Regional Classification Scheme for Estimating Reference Water Quality in Streams Using Land-Use-Adjusted Spatial Regression-Tree Analysis. *Environ. Manage.* **37**, 209-229 (2006).
66 Smith, R. A., Alexander, R. B. & Schwarz, G. E. Natural Background Concentrations of Nutrients in Streams and Rivers of the Conterminous United States. *Environ. Sci. Technol.* **37**, 3039-3047 (2003).
67 Hawkins, C. P., Olson, J. R. & Hill, R. A. The reference condition: predicting benchmarks for ecological and water-quality assessments. *J. N. Am. Benthol. Soc.* **29**, 312-343 (2010).
68 Stoddard, J. L., Larsen, D. P., Hawkins, C. P., Johnson, R. K. & Norris, R. H. Setting expectations for the ecological condition of streams: the concept of the reference condition. *Ecol. Appl.* **16**, 1267-1276 (2006).
69 Lehner, B. & Grill, G. Global river hydrography and network routing: baseline data and new approaches to study the world's large river systems. *Hydrol. Process.* **27**, 2171-2186 (2013).
